# Comparative Analysis of Neuropeptides in Homologous Interneurons and Prohormone Annotation in Nudipleuran Sea Slugs

**DOI:** 10.3389/fphys.2021.809529

**Published:** 2021-12-23

**Authors:** Colin A. Lee, Elena V. Romanova, Bruce R. Southey, Rhanor Gillette, Jonathan V. Sweedler

**Affiliations:** ^1^Neuroscience Program, University of Illinois Urbana-Champaign, Urbana, IL, United States; ^2^Department of Chemistry, University of Illinois Urbana-Champaign, Urbana, IL, United States; ^3^Department of Animal Sciences, University of Illinois Urbana-Champaign, Urbana, IL, United States; ^4^Department of Molecular and Integrative Physiology, University of Illinois Urbana-Champaign, Urbana, IL, United States

**Keywords:** mass spectrometry, bioinformatics, peptidomics, neuroethology, mollusk, invertebrate, evolution

## Abstract

Despite substantial research on neuronal circuits in nudipleuran gastropods, few peptides have been implicated in nudipleuran behavior. In this study, we expanded the understanding of peptides in this clade, using three species with well-studied nervous systems, *Hermissenda crassicornis*, *Melibe leonina*, and *Pleurobranchaea californica*. For each species, we performed sequence homology analysis of *de novo* transcriptome predictions to identify homologs to 34 of 36 prohormones previously characterized in the gastropods *Aplysia californica* and *Lymnaea stagnalis*. We then used single-cell mass spectrometry to characterize peptide profiles in homologous feeding interneurons: the multifunctional ventral white cell (VWC) in *P. californica* and the small cardioactive peptide B large buccal (SLB) cells in *H. crassicornis* and *M. leonina*. The neurons produced overlapping, but not identical, peptide profiles. The *H. crassicornis* SLB cells expressed peptides from homologs to the FMRFamide (FMRFa), small cardioactive peptide (SCP), LFRFamide (LFRFa), and feeding circuit activating peptides prohormones. The *M. leonina* SLB cells expressed peptides from homologs to the FMRFa, SCP, LFRFa, and MIP-related peptides prohormones. The VWC, previously shown to express peptides from the FMRFa and QNFLa (a homolog of *A. californica* pedal peptide 4) prohormones, was shown to also contain SCP peptides. Thus, each neuron expressed peptides from the FMRFa and SCP families, the *H. crassicornis* and *M. leonina* SLB cells expressed peptides from the LFRFa family, and each neuron contained peptides from a prohormone not found in the others. These data suggest each neuron performs complex co-transmission, which potentially facilitates a multifunctional role in feeding. Additionally, the unique feeding characteristics of each species may relate, in part, to differences in the peptide profiles of these neurons. These data add chemical insight to enhance our understanding of the neuronal basis of behavior in nudipleurans and other gastropods.

## Introduction

Due to their relatively simple nervous systems and individually identifiable neurons, nudipleuran gastropods have yielded considerable insight into the neuronal basis of behavior (Katz and Quinlan, 2019). The neuronal circuits governing certain behaviors are well described (Elliott and Susswein, 2002; Crow, 2004; Gillette and Brown, 2015; Sakurai and Katz, 2015), yet there is relatively little information on the peptides and hormones regulating these circuits. Only one nudipleuran, *Tritonia diomedea*, has been the subject of a broad scale peptidomic study (Senatore et al., 2015) and physiological studies have largely focused on only three peptides: pedal peptide (Beck et al., 2000; Baltzley et al., 2011), FMRFamide (FMRFa; Lillvis et al., 2012; Webber et al., 2017), and one of the small cardioactive peptides (SCP; Watson and Willows, 1992; Lillvis et al., 2012; Watson et al., 2020). Because 100s of peptides and hormones are present in many species (Ma et al., 2009; Cafe-Mendes et al., 2014; Gan et al., 2015; Christie and Pascual, 2016; Van Camp et al., 2017), and peptidergic signaling is diverse and essential to animal behavior (Liu et al., 2008), the limited peptidomic insight constitutes a considerable gap in our understanding of nudipleuran physiology. Research on other gastropods further highlights this gap. Numerous peptides and peptide prohormones have been identified in the gastropods *Aplysia californica* and *Lymnaea stagnalis* (Hummon et al., 2003b; Di Cosmo and Di Cristo, 2006; Feng et al., 2009; Cropper et al., 2018; Wood et al., 2021), and recent large-scale transcriptomic studies have examined peptides in the snails *Theba pisana* (Adamson et al., 2015), *Deroceras reticulatum* (Ahn et al., 2017), and *Charonia tritonis* (Bose et al., 2017). Identifying more peptides in nudipleurans will help to rectify this deficit.

Nudipleurans also permit analysis of individual, homologous neurons, that is, neurons with conserved neuroanatomy and function (Bulloch and Ridgway, 1995; Sakurai and Katz, 2019). By comparing homologous neurons one can examine the evolution of behavior at the level of a single neuron (Croll, 1987), and moreover, enables examination of traits at the level of a clade rather than a single species, revealing trends in specific traits (Jourjine and Hoekstra, 2021). In most species, it is difficult to reproducibly identify individual neurons, but nudipleurans, with their large neuronal cell bodies, simple behaviors, and relatively few neurons (less than 10,0000; Boyle et al., 1983), allow for such identification (Katz and Quinlan, 2019). Additionally, the large neuronal sizes are well suited for single-cell peptidomic analysis, performed using matrix-assisted laser desorption/ionization (MALDI)-time-of-flight (TOF) mass spectrometry (MS; Garden et al., 1996; Li et al., 2000a). Several homologous neurons have been characterized across nudipleurans (Baltzley et al., 2011; Lillvis et al., 2012; Newcomb et al., 2012), and detailed analyses have uncovered the subtle differences in their circuit-level roles (Katz, 2016; Sakurai and Katz, 2019). Characterizing the peptide profiles of homologous neurons is a logical next step to this research.

This study characterized peptides in both homologous neurons and central nervous system (CNS) transcriptomes for three nudipleuran sea slugs, *Hermissenda crassicornis*, *Melibe leonina*, and *Pleurobranchaea californica*. Each species has been the subject of extensive neurophysiological research (Crow and Tian, 2006; Sakurai et al., 2014; Gillette and Brown, 2015), has a publicly deposited CNS transcriptome, and an easily identified, homologous feeding interneuron in its buccal ganglion. In each species, this neuron drives fictive feeding rhythms and extends axons to extensively innervate the esophagus. In *P. californica*, this cell, the ventral white cell (VWC), has been shown to be multifunctional (Gillette et al., 1980; Gillette and Gillette, 1983), playing both a command and a motor role in feeding behavior. In *H. crassicornis* and *M. leonina,* these are the SCP_B_ large buccal (SLB) cells, which stain for SCP_B_ (Watson and Willows, 1992). Furthermore, each species has unique feeding characteristics that can be correlated with peptide identities. *M. leonina* lacks a buccal mass or radula for food breakdown, is a filter feeder, engages in hours-long feeding bouts, and has only approximately 40 neurons in its buccal ganglion (Watson and Trimarchi, 1992; Lee and Watson, 2016). *H. crassicornis* feeds primarily on polyps and jellyfish (Hoover et al., 2012), and *P. californica* is an active, generalist predator that indulges in cannibalism (Noboa and Gillette, 2013). These species thus allow for a meaningful comparison of homologous interneurons, using existing resources for rapid annotation of their transcriptomes.

For prohormone annotation, we obtained the CNS *de novo* transcriptome assemblies because no genomic assemblies for *H. crassicornis*, *M. leonina*, and *P. californica* are available. Using 36 known *A. californica* and *L. stagnalis* prohormones, we identified homologous prohormones within each species, and from these compiled a library of putative encoded peptides for each species. We then performed single-cell MS on individual VWCs and SLB cells and used the peptide library to perform peptide mass fingerprinting (Thiede et al., 2005) on the resulting spectra. In *H. crassicornis* we detected peptides from homologs to the FMRFa, SCP, LFRFamide (LFRFa), and feeding circuit activating peptides (FCAP) prohormones, and in *M. leonina*, peptides from homologs to the FMRFa, SCP, LFRFa, and myoinhibitory peptide (MIP)-related prohormones. The *P. californica* VWC has previously been shown to have peptides from the FMRFa and QNFLa [a homolog of the *A. californica* pedal peptide 4; (Green et al., 2018)] prohormones, and we found that it also contains peptides from the SCP prohormone. Thus, each species’ neuron expressed peptides from both the FMRFa and SCP prohormones, and both *H. crassicornis* and *M. leonina* expressed peptides from the LFRFa prohormone; however, each neuron also expressed peptides not seen in the others. This work provides an untargeted peptidomic characterization of single homologous neurons and a large-scale prohormone annotation of multiple nudipleuran sea slugs.

## Materials and Methods

### Animal Care

*P. californica* and *H. crassicornis* were trapped by the Monterey Abalone Company (Monterey Bay, CA) and shipped overnight to the University of Illinois Urbana-Champaign. There they were housed individually in artificial seawater at 12°C. *M. leonina* were collected at the San Juan Islands, either off the docks of Friday Harbor Labs (San Juan, WA), or *via* snorkel/scuba diving at Park’s Bay (Shaw Island, WA), and maintained at Friday Harbor Labs in sea tables with flow-through sea water.

### *In silico* Prohormone Annotation and Peptide Library Establishment

For each species, prohormone annotations were performed on publicly available *de novo* RNA transcriptome assemblies from the NCBI Sequence Read Archive (Kodama et al., 2012; Christie, 2017; Southey et al., 2020). Species-specific information is as follows: *H. crassicornis* – SRR1719366 (Goodheart et al., 2017), *M. leonina* – SRR1950947 and SRR3738852 (Goodheart et al., 2017), and *P. californica* – SRR026692, SRR026693, SRR026694, SRR026695, SRR1505130, and SRR3928990 (Zapata et al., 2014). For each experimental data set, *de novo* assemblies were created without any preprocessing of reads using MEGAHIT (Li et al., 2015), SOAPdenovo (Luo et al., 2012), and Trinity (Grabherr et al., 2011; Haas et al., 2013) with default settings. *De novo* assemblies from the same species were combined into a single BLAST (Altschul et al., 1997) database.

For each species, A TBLASTN search was performed on a database of *de novo* assemblies for 34 *A. californica* and two *L. stagnalis* neuropeptide prohormones obtained from the UniProt database (Apweiler et al., 2004), yielding both RNA and protein matches. RNA matches were translated using the ExPaSy “Translate” tool (Gasteiger et al., 2003), and the longest predicted protein sequence from the matched region was selected for further analysis. SignalP 5.0 (Armenteros et al., 2019) and Phobius (Kall et al., 2007) were then used to analyze both translated proteins and direct protein sequences for the presence of a signal sequence, which is required for targeting into the secretory pathway (Rusch and Kendall, 1995). Finally, potential neuropeptides from each matching protein, whether complete (i.e., possessing a signal sequence) or not, were predicted using NeuroPred (Southey et al., 2006a,b, 2008) with the Mollusc model (Hummon et al., 2003a) and common PTMs selected. The resulting predicted peptides were compiled to form a putative peptide library for each species.

### Single-Cell Isolation and MALDI-TOF MS Analysis

We followed prior approaches (Li et al., 2000a) for single-cell isolation and MALDI MS characterization. Subjects were pinned out in dissecting trays, and buccal ganglia were surgically removed. Ganglia were then incubated for 6 min in 1% type 14 protease prepared in saline (460 mm NaCl, 10 mm KCl, 10 mm CaCl_2_, 25 mm MgCl_2_, 25 mm MgSO^4−^, 10 mm HEPES, pH = 7.6), which loosened the surrounding connective sheath. Moria scissors were then used to cut through the connective sheath (one layer in *M. leonina* and *H. crassicornis*, two layers in *P. californica*) to expose the neurons. Neurons were identified visually by their distinct morphology, color, and landmark location within the ganglion (Gillette et al., 1980; Watson and Willows, 1992), and then carefully teased away from the rest of the ganglion using either pulled glass capillaries or tungsten needles. Isolated neurons were then aspirated into a custom-made transfer pipette and spotted onto a ground steel MALDI sample plate (Bruker Corp., Billerica, MA), and 0.5 μl of matrix solution (dihydroxybenzoic acid, 20 mg/ml in deionized water) was applied to each neuron. Following drying and matrix crystallization, samples were analyzed by MALDI-TOF MS using an ultrafleXtreme mass spectrometer (Bruker Corp.) in positive reflectron mode, with a surveyed mass range of 530–5,000 *m/z* and external calibration. Once the spectra had been collected, detected masses were matched to those in the peptide libraries by peptide mass fingerprinting (Thiede et al., 2005) with an allowed mass match error of 200 ppm.

## Results

### *In silico* Transcriptomic Annotation of Putative Neuropeptide Prohormones

*De novo* transcriptomes of the three nudipleurans were queried against 34 *A. californica* and two *L. stagnalis* neuropeptide prohormones to identify 35 transcripts in *H. crassicornis* and *M. leonina*, and 34 *P. californica* (Table 1; Supplementary Tables S1–S3). This included two SCP prohormones in *H. crassicornis* and *M. leonina*, and two *M. leonina* temptin proteins. Two proteins, *A. californica* attractin and egg-laying hormone, were searched but did not yield matches in any of the three species. The majority (27 in *H. crassicornis*, 27 in *M. leonina*, and 26 in *P. californica*) contained a predicted signal sequence. However, only 19, 17, and 12 transcripts of *H. crassicornis*, *M. leonina,* and *P. californica*, respectively, had sequence lengths of at least 95% of *A. californica* and *L. stagnalis* neuropeptide prohormone sequence lengths. Each prohormone encoded peptides homologous to those found in the *A. californica*/*L. stagnalis* versions of the prohormone.

**Table 1:** Prohormone annotations of each species’ CNS transcriptome.

Name	Query			*H. crassicornis*				*M. leonina*				*P. californica*			
	Accn	Organism	nR	% Id	Evalue	Signal length	nR	% Id	Evalue	Signal length	nR	% Id	Evalue	Signal length	nR
Abdominal ganglion neuropeptide L11	P06518	*Aplysia californica*	151	8.1	0.756	25	161	15.2	0.000353		112	14.3	3.00E-04	27	175
Abdominal ganglion neuropeptide L5-L67	P07712	*A. californica*	112	33.9	3.35E-20	22	124	35.4	9.00E-10	23	124	37.9	1.42E-18	23	103
Abdominal ganglion neuropeptide R3-14	P01364	*A. californica*	108	21.9	5.18E-06	23	114	21.9	8.06E-06	23	114	23.4	5.7	25	77
Achatin	Q5MAR6	*A. californica*	158	31.3	5.14E-05	24	131	52	8.74E-06		98	25.4	1.87	22	134
Adipokinetic hormone (AKH)	I6YDN8	*A. californica*	80	29.7	8.17E-09	31	91	30.2	1.22E-08	23	86	30.9	2.42E-07	22	81
Atrial gland and califin peptides	P01360	*A. californica*	173	21.3	0.0521		94	8.8	0.0132	29	306	14.4	1.22		153
Buccalin	P20481	*A. californica*	505	35.4	3.63E-60	24	328	34.8	2.34E-52	26	302	48.6	4.75E-96		368
Cerebral Peptide	Q10998	*A. californica*	209	54.1	1.53E-52	19	194	47.9	3.00E-15	30	194	28.2	5.45E-09	30	142
Cerebrin	Q8T112	*A. californica*	86	30.3	9.93E-11	23	89	48.4	3.00E-08	28	95	20.7	0.000116	25	82
Enterin	Q95P23	*A. californica*	837	44.7	1.02E-43		215	46.4	1.5	25	414	48	9.59E-57		323
Enticin	Q8I817	*A. californica*	88	33.3	5.48		33					31.6	3.37		57
Feeding circuit activating peptides (FCAP)	Q8ISH7	*A. californica*	504	31.5	6.23E-92	22	743	74.8	5.00E-06	17	216	48.8	4.70E-86	26	404
FMRF-amide neuropeptide	P08021	*A. californica*	597	27.7	2.46E-23	23	386	22.6	5.00E-20	25	285	15.1	5.34E-19	23	885
FMRFa-related neuropeptides	P42565	*L. stagnalis*	360	49.4	9.15E-21		154	21.9	7.89E-31	25	430	40.2	3.32E-70	24	338
Gonadotropin-releasing hormone (GNRH)	A8WA77	*A. californica*	147	36.3	1.13E-11	23	124	29	3.00E-09		162	27.9	1.56E-05	34	104
Insulin	Q9NDE7	*A. californica*	156	33.3	2.69E-21	29	168	32.2	2.30E-21	33	149	46.1	2.35E-22	31	141
LFRF	Q5U900	*L. stagnalis*	194	38	1.62E-15	22	208	37.5	1.00E-16	22	176	41.2	2.11E-19	22	215
MIP-related peptides	Q9NDE8	*A. californica*	735	35.3	6.05E-26		272	31.9	4.00E-12		668	9.7	6.97E-12		444
Myomodulin 1	P15513	*A. californica*	370	32.4	4.30E-42	21	426	58.1	0.53	16	277	37.5	2.06E-41	21	344
Myomodulin 2	Q2VF17	*A. californica*	240	28.8	1.01E-14	25	187	41.4	6.00E-14	22	215	33.2	3.06E-20	25	244
NdWFamide	A0A161R9R0	*A. californica*	90	45.9	6.77E-11	25	83	45.8	9.75E-11	25	83	40.5	6.22E-10	25	84
Neuroactive Polyprotein R15	P12285	*A. californica*	156	21.2	1.01E-08	28	151	33.5	0.95	28	170	34.5	1.50E-05		84
Neuro-peptides CP2	Q8T0Y7	*A. californica*	141	14	0.00217	23	129	9.5	2.412	23	126	18	2.59	24	128
NPY	Q27441	*A. californica*	92	45.7	3.14E-22	21	92	52.7	9.00E-18	21	91	35.6	3.88E-13	21	90
Pedal Peptide 1	Q5PSJ2	*A. californica*	385	38.8	2.77E-113	18	418	11	3.25E-13	18	281	51.7	4.49E-47	20	174
Pedal Peptide 2	A1XP49	*A. californica*	628	52.4	2.47E-47	21	203	55.2	0		645	27.7	9.74E-43	34	476
Pedal Peptide 3	A1XP50	*A. californica*	307	34.1	1.59E-56	21	323	35.7	3.61E-45	23	249	43.8	4.72E-39	20	256
Pedal Peptide 4	A1XP51	*A. californica*	535	27.5	5.84E-22		444	42.3	8.00E-13		478	25.2	1.03E-75	34	782
Pleurin	Q5PSJ5	*A. californica*	188	35.1	1.41E-24	19	205	35.7	2.00E-26	19	196	44.9	2.36E-32	18	176
PRQFV	Q86MA7	*A. californica*	862	32.6	2.26E-56		763	28.8	4.00E-22	23	319	53.9	3.59E-95		495
Small cardioactive peptides 1	P09892	*A. californica*	136	43.9	1.09E-35	24	132	49.6	2.00E-27	24	141	57	3.82E-31	25	135
Small cardioactive peptide 2	P09892	*A. californica*	136	58.3	1.19E-34	24	132	62	3.23E-35	24	122				
Sensorin A	P29233	*A. californica*	113	38.5	2.40E-16	31	117	34.4	3.00E-16	30	160	30.4	7.04E-16	29	115
Temptin 1	Q7Z0T3	*A. californica*	125	27.3	4.76E-20	25	128	44.6	9.00E-33	40	157	29.5	1.63E-33	22	190
Temptin 2	Q7Z0T3	*A. californica*	125					52.3	3.00E-31	16	100				
Whitnin	Q5PSJ3	*A. californica*	116	54.7	1.96E-38	23	117	54.7	9.01E-40	23	117	0.5	6.49E-36	23	116

*A. californica* or *L. stagnalis* versions of each prohormone were searched against de novo transcriptome assemblies for each species’ CNS. Accn – accession number. nR – number of amino acid residues in protein. % Id – degree to which the two prohormones have the same residue at the same point in the alignment. Evalue – likelihood of achieving a comparable match by chance. Signal length – length of signal peptide. nR – number of amino acid residues in returned prohormone.

### Identification of Novel Small Cardioactive Peptide C

**Figure 1:**
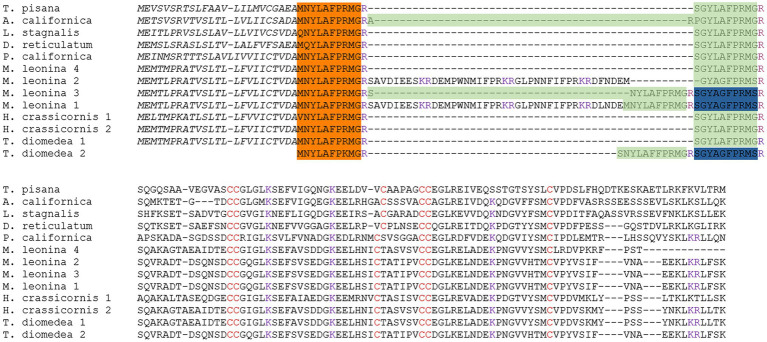
Multiple sequence alignment of SCP prohormones across species and predicted protein isoforms. *Melibe leonina* yielded four SCP prohormone isoforms, and *Hermissenda crassicornis* and *Tritonia diomedea* each yielded two. Each prohormone encoded SCP_B_ (orange highlight) and SCP_A_ (green highlight), and one *T. diomedea* and two *M. leonina* isoforms encoded SCP_C_ (blue highlight). Conserved cysteine (red font) and predicted or known cleavage sites (purple font) are also indicated.

Further analysis of the transcriptomes found two protein isoforms for the SCP prohormone in *H. crassicornis* and four in *M. leonina* (Figure 1). The *M. leonina* isoforms all expressed identical signal sequences and both SCP_A_ and SCP_B_ neuropeptides, and three of the four shared the same 94 C-terminal amino acids. Two of the isoforms also expressed a novel peptide, SCP_C_, which differed from the other SCPs in that it has a serine at the C-terminus, lacked amidation, and is 10 amino acids long rather than nine. However, it retains the YXXFPRM motif seen in all other SCPs, including those found in *A. californica* (P09892), *L. stagnalis* (O97374), the snail *T. pisana* (A0A0S1RSH0), and the snail *D. reticulatum* (A0A1X9WEF6; Figure 1). Although SCP_C_ has not been observed in any of the above species, it was identified in one of two SCP protein isoforms in *T. diomedea*.

### Unique Neuropeptide Profiles of SLB Cells and VWC Among Nudipleuran Species

**Figure 2:**
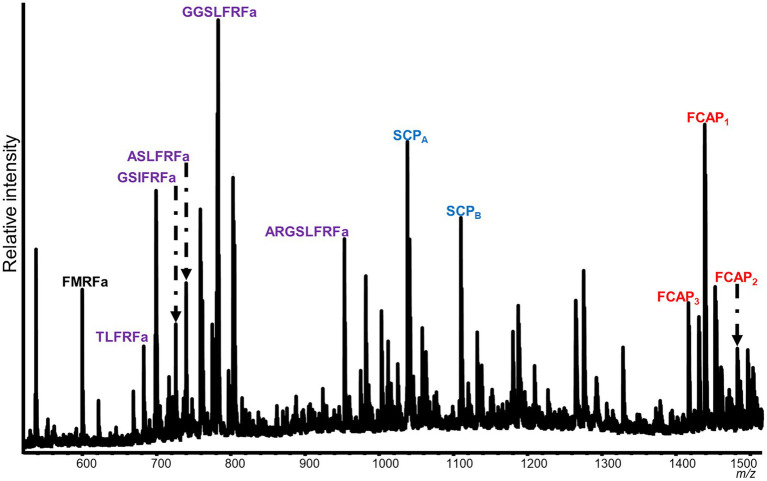
Representative spectrum from a *H. crassicornis* SLB cell. Six neurons were analyzed, and peptides from the FMRFa (black), SCP_B_ (blue), LFRFa (purple), and FCAP (red) prohormones were present. The LFRF prohormone is predicted to produce five peptides with a C-terminal LFRFa motif, the FCAP prohormone, three versions of the FCAP peptide, and the SCP prohormone, both SCP_A_ and SCP_B_; all of these were present in the cell. The FMRFa prohormone is predicted to produce multiple tetrapeptides, but only FMRFa was present in the cell.

**Figure 3:**
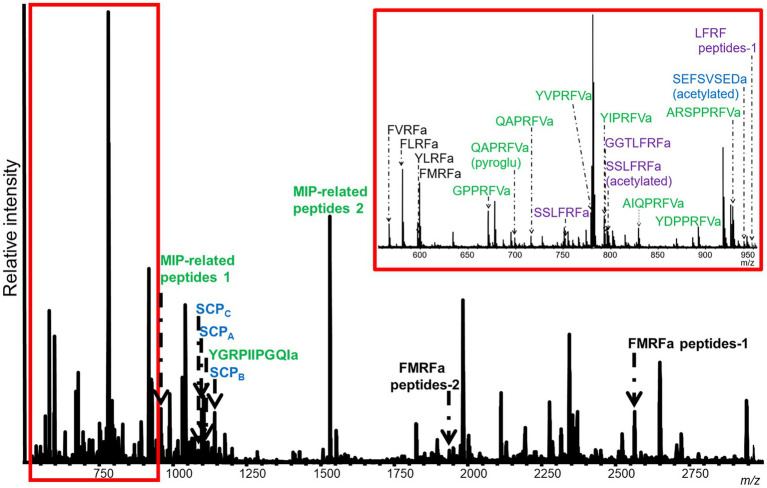
Representative MALDI-TOF MS spectrum from a *M. leonina* SLB cell. Four neurons were analyzed. This cell expressed 11 peptides from the MIP-related prohormone (green), six peptides from the FMRFa prohormone (black), and four peptides from the SCP (blue) and LFRFa (purple) prohormones. Peptides from the MIP-related prohormone included eight, with either a PRFVa or PTFVa C-terminal motif. The peptides from the SCP prohormone included SCP_A_, SCP_B_, and SCP_C_. Four of the six peptides from the FMRFa were tetrapeptides, including FMRFa itself. Lastly, the peptides from the LFRFa prohormone included both an acetylated and unacetylated version of SSLFRFa.

Mass spectrometric analysis of individual SLB cells found that the peptides SCP_A_ and SCP_B_, encoded by species-specific homologues of the *A. californica* SCP prohormone (UniProt accession number: P09892), were present (Figures 2, 3). The *M. leonina* SLB cells contained two other peptides encoded by the SCP prohormone (GGCA01092244.1), including SCP_C_. In addition, peptides encoded by several other neuropeptide genes co-localized in the SLB cells, although combinations differed by species (Table 2). The *H. crassicornis* SLB cells contained FMRFa and peptides from homologs to the *L. stagnalis* LFRFa prohormone (Q5U900) (Supplementary Figure S1) and *A. californica* FCAP prohormone (Q8ISH7) (Supplementary Figure S2). The *H. crassicornis* LFRFa prohormone encodes five different peptides with a conserved LFRFa motif and amidated C-terminus (Supplementary Figure S1), and each was present within the *H. crassicornis* SLB cells. Finally, the *H. crassicornis* FCAP prohormone encoded three structurally similar peptides, and each was confirmed by MS in the neuron.

**Table 2:** MALDI-TOF MS identification of neuropeptides in SLB cells/VWCs of nudipleurans.

Species	Prohormone family homology	Peptide sequence	Peptide name	Mean M + H	Theoretical M + H	Mass error ppm
*H. crassicornis*	FCAP	GLDSLGGFNVHGGW	FCAP_3_	1415.684	1415.668	11.3
	FCAP	GLDSLGGFQVHGGW	FCAP_1_	1429.71	1429.684	18.2
	FCAP	GLDSLGGFHVHGGGW	FCAP_2_	1495.7	1495.706	-4
	FMRFa	FMRFamide	FMRFa	599.279	599.312	−55.1
	LFRFa	TLFRFamide	TLFRFa	682.393	682.403	−14.7
	LFRFa	GSIFRFamide	GSIFRFa	725.404	725.409	−6.9
	LFRFa	ASLFRFamide	ASLFRFa	739.423	739.425	−2.7
	LFRFa	GGSLFRFamide	GGSLFRFa	782.464	782.431	42.2
	LFRFa	ARGSLFRFamide	ARGSLFRFa	952.594	952.547	49.3
	SCP	SGYLAFPRMamide	SCP_A_	1041.588	1041.535	50.9
	SCP	VNYLAFPRMamide	SCP_B_	1109.638	1109.592	41.5
*M. leonina*	FMRFa	FVRFamide	FVRFa	567.313	567.34	−47.6
	FMRFa	FLRFamide	FLRFa	581.338	581.356	−31
	FMRFa	YLRFamide	YLRFa	597.335	597.35	−25.1
	FMRFa	FMRFaide	FMRFa	599.303	599.312	−15
	FMRFa	RSVDDDDMSTRSGDVID	FMRFa peptides-2	1882.809	1882.806	1.6
	FMRFa	SQQPNVDDIYNKALLQLEEPYS	FMRFa peptides-1	2564.237	2564.249	−4.7
	LFRFa	SSLFRFamide	SSLFLRa	755.442	755.42	29.1
	LFRFa	GGTLFRFamide	GGTLFRFa	796.477	796.446	38.9
	LFRFa	acSSLFRFamide	SSLFRFa (acetylated)	797.432	797.43	2.5
	LFRFa	acSGPQSNEGM	LFRF peptides-2	948.51	948.371	146.6
	MIP-related	GPPRFVamide	GPPRFVa	671.414	671.398	23.8
	MIP-related	pQAPRFVamide	QAPRFVa (pyroglutamated)	699.411	699.393	25.7
	MIP-related	QAPRFVamide	QAPRFVa	716.442	716.42	30.7
	MIP-related	YVPRFVamide	YVPRFVa	779.493	779.456	47.5
	MIP-related	YIPRFVamide	YIPRFVa	793.511	793.472	49.2
	MIP-related	AIQPRFVamide	AIQPRFVa	829.546	829.504	50.6
	MIP-related	YDPPRFVamide	YDPPRFVa	892.514	892.4673	52.3
	MIP-related	ARSPPRFVamide	ARSPPRFVa	928.594	928.54726	50.3
	MIP-related	acGPSLQASEE	MIP-related peptides 1	959.519	959.43	92.8
	MIP-related	YGRPIIPGQIamide	YGRPIIPGQIa	1112.707	1112.6572	44.8
	MIP-related	DYDTIFDLLHNSA	MIP-related peptides 2	1523.721	1523.699	14.4
	SCP	acSEFSVSEDamide	SCP peptides-1	940.5413	940.389	162
	SCP	SGYAGFPRMS	SCP_C_	1072.541	1072.486	51.3
	SCP	SNYLAFPRMamide	SCP_A_	1097.605	1097.556	44.6
	SCP	MNYLAFPRMamide	SCP_B_	1141.616	1141.564	45.6
*P. californica*	SCP	SGYLAFPRMamide	SCP_A_	1041.6403	1041.535	101.1
	SCP	MNYLAFPRMamide	SCP_B_	1141.7383	1141.564	152.7
	FMRFa	ASAGGQRSEESLLREALMQAEEPLY	AEEPLY	Previously characterized		
	FMRFa	SEESLLREALMQAEEPLY	AEEPLY’			
	FMRFa	FLRFamide	FLRFa			
	FMRFa	FMRFamide	FMRFa			
	FMRFa	DVGGGSAAGDAEEEDIISRQILGLGGGQVGESGDVIDGF	FMRFa peptide 3			
	FMRFa	PSNAALEGLEGE	FMRFa peptide 5			
	QNFLa	(p-)QLDSIGAGMVSGLHQNFL(Amide)	QNFLa-peptide 5			
	QNFLa	FDSISSGRLNGFNANFL(Amide)	QNFLa-peptide 6			

Six SLB cells analyzed from *H. crassicornis*, four SLB cells from *M. leonina*, and five VWCs from *P. californica*. PPM – parts per million. Both mean and theoretical M + H values are the average molecular weight.

**Figure 4:**
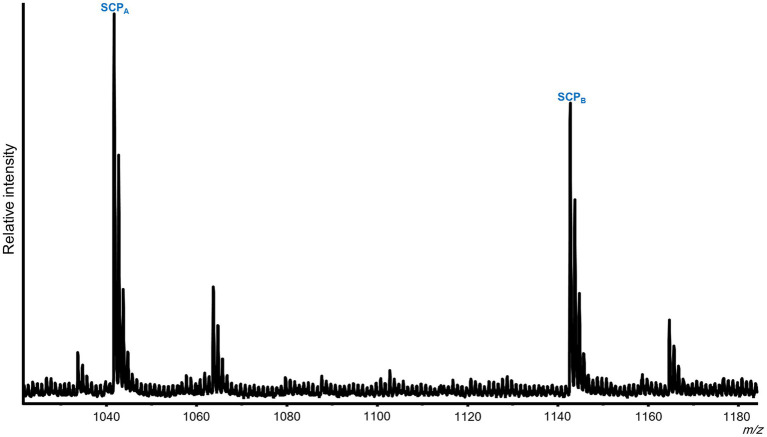
Representative MALDI-TOF MS spectrum from a *Pleurobranchaea californica* VWC. Five neurons were analyzed. In addition to the previously described peptides from the FMRFa and QNFLa prohormones (Green et al., 2018), the VWC expressed both SCP_A_ and SCP_B_.

In addition to peptides from the SCP prohormone, the *M. leonina* SLB cells contained peptides matching three other prohormones (Figure 3): a homolog to the *A. californica* MIP-related prohormone (Q9NDE8) (Supplementary Figure S3), a homolog to the *A. californica* FMRFa prohormone (P08021) (Supplementary Figure S4), and a homolog to the *L. stagnalis* LFRFa prohormone (Supplementary Figure S1). Eleven peptides derived from the MIP-related prohormone, including eight with a C-terminal amidation and a PRFV or PTFV motif, similar to the PRFX motif found in the *A. californica* MIP-related prohormone. Six peptides came from the FMRFa prohormone, including FMRFa and three other -RFa tetrapeptides, and four peptides from the LFRFa prohormone (Table 2). Two of these four had the same amino acid sequence (GGTLFRF), differing only in the post-translational addition of an acetyl group, and a third peptide also shared the LFRFa motif. Interestingly, two other peptides with an LFRFa motif were putatively encoded on the *M. leonina* LFRFa prohormone, yet were not detected in the SLB cells by MS. Finally, the *P. californica* VWC also contained both SCP_A_ and SCP_B_ (Figure 4; Table 2).

## Discussion

### *De novo* Assembly of CNS Transcriptomes

Peptides in nudipleurans remain understudied and yet have been extensively studied in the gastropods *A. californica* and *L. stagnalis*. Exploring peptides in nudipleurans can enhance the existing understanding of their neuronal circuits while also allowing for comparison with other species, an essential task for the study of brain evolution (Webber et al., 2017; Moroz, 2018). Using *de novo* transcriptome assemblies, we predicted putative peptide prohormones for *H. crassicornis*, *M. leonina*, and *P. californica*, and identified several homologs to prohormones previously characterized in *A. californica* and *L. stagnalis*. Additionally, in *M. leonina* we identified alternatively spliced SCP transcripts that encoded a novel peptide. Although we recovered homologs to almost every searched transcript, in some instances we could not recover the full protein sequence, and thus it is possible that we missed certain peptides. It is also possible that the transcripts not found here are indeed present in these species, but simply not expressed in the tissues used to generate the transcriptome assemblies.

### Peptide Profile Diversity of the VWC and SLB Cells in Nudibranchs, and Functional Implications

**Figure 5:**
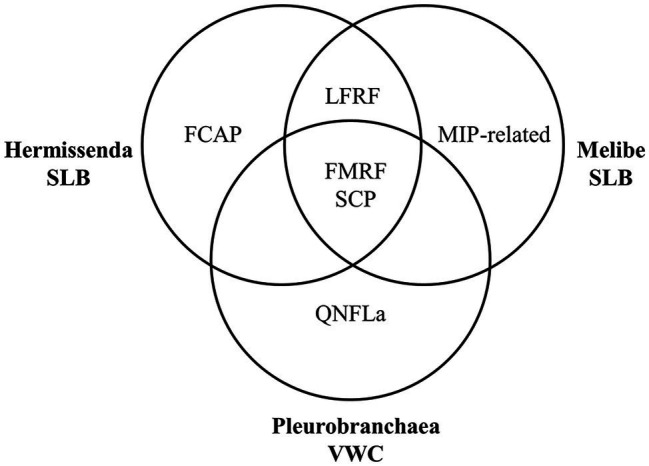
Summary of the prohormone families present in each species’ neuron. Each species has peptides from the FMRFa and SCP prohormone families, both nudibranchs have peptides from the LFRFa prohormone family, and each species has a prohormone family not detected in the other species’ neurons.

We found that the neuropeptide complements of the VWC and SLB cells overlapped but were not identical (Figure 5), which may reflect adaptation to the species’ different feeding habits. SCP peptides were present in every cell, consistent with earlier immunological work (Watson and Willows, 1992) and suggesting a conserved role for these peptides in feeding. In *P. californica*, VWC firing drives esophageal dilation (Gillette and Gillette, 1983), and in *M. leonina*, SCP_B_ application causes esophageal contractions (Watson et al., 2020), so the data suggest that these neurons use SCP_B_ to regulate esophageal movement.

Meanwhile, SCPs are extensively involved in the control of feeding motor programs in *A. californica* (Lloyd, 1986; Lloyd et al., 1987) and *L. stagnalis* (Santama et al., 1994; Perry et al., 1999). The *A. californica* B1 and B2 and the *L. stagnalis* B2 neurons contain SCP_A_ and SCP_B_ and project axons to the esophageal nerve (Lloyd et al., 1988; Santama et al., 1994; Perry et al., 1998, 1999), and additional *A. californica* SCP-immunoreactive neurons innervate buccal musculature (Lloyd, 1988; Church et al., 1991). In both species, the SCPs co-localize with each other (Perry et al., 1998; Perry et al., 1999; Li et al., 2000b), and act as co-transmitters with both classical neurotransmitters (Weiss et al., 1992; Perry et al., 1999) and other neuropeptides (Santama et al., 1994). SCP also drives rhythmic bursting in the buccal ganglion of the snail *Helisoma trivolvis* (Murphy et al., 1985) and has even been implicated in feeding in *Octopus vulgaris*, as it drives contraction of the radula protractor muscle, and is transcribed in the buccal ganglion (Kanda and Minakata, 2006). The studies discussed here are insufficient to determine if *A. californica* and *L. stagnalis* have homologs to the VWC/SLB cells, but a clear conserved role for SCP can be seen in feeding-related movements.

FMRFa was previously found in the VWCs (Green et al., 2018) and was also found here within the SLB cells, which was surprising given that it inhibits feeding in other gastropods. In *A. californica*, the FMRFa peptide partially shifts feeding rhythms from ingestive to egestive and is released from sensory neurons to reduce accessory radula closer (ARC) muscle contractions (Vilim et al., 2010). Meanwhile, in *L. stagnalis* (Kyriakides and McCrohan, 1989) and *H. trivolvis* (Murphy et al., 1985), FMRFa perfusion inhibits the feeding rhythm, although in *L. stagnalis* it appears to be released from a pleural interneuron involved in defensive responses (Alania et al., 2004), rather than from an element of the feeding neural network. The *L. stagnalis* buccal mass is immunopositive for FMRFa and the buccal ganglion contains a single, bilateral neuron pair with immunoreactivity to the related peptide SEQPDVDDYLRDWLQSEEPLY (Santama et al., 1994), but FMRFa itself has not been detected in the *L. stagnalis* buccal ganglion by MS. Meanwhile, numerous sensory and motor neurons express FMRFa in the *A. californica* buccal ganglion (Vilim et al., 2010). Does the FMRFa released from the VWC/SLB cells in some way attenuate feeding, or does the presence of FMRFa in these cells reflect divergence from its role in *A. californica* and *L. stagnalis*? Our finding opens the door for future functional studies to address this question.

LFRFa peptides were observed in the *H. crassicornis* and *M. leonina* SLB cells but not the *P. californica* VWC, a pattern that perhaps reflects the three species’ phylogeny. The nudipleuran clade separates into nudibranchia and pleurobranchomorpha; *H. crassicornis* and *M. leonina* are nudibranchs whereas *P. californica* is a pleurobranch. In *A. californica*, LFRFa peptides have a similar effect as FMRFa, modulating contraction of the ARC muscle and weakening ingestive feeding rhythms (Cropper et al., 1994; Vilim et al., 2010). In *L. stagnalis*, MS analysis of the buccal ganglion found the presence of the six peptides encoded by the LFRFa prohormone, which inhibit neurons that regulate metabolism (Hoek et al., 2005). However, immunostaining and single-cell analysis have not been carried out thus far, nor is it known if these peptides have a role in *L. stagnalis* feeding circuitry. Thus, it will be of interest to determine the roles of LFRFa peptides in feeding in other species. Similarly, it will be of interest will be to determine if other cells within the *P. californica* buccal ganglion express LFRFa.

Finally, each species’ neuron expressed peptides from a prohormone not detected by MS in the others. First, the *H. crassicornis* SLB cells contained three peptides from the homolog to the *A. californica* FCAP prohormone and may contain more, as our annotation of the *H. crassicornis* FCAP prohormone returned an incomplete protein. FCAP drives feeding rhythms in *A. californica*, and interestingly is co-expressed with SCP_B_ in a mechanosensory neuron (Sweedler et al., 2002), but its effects on feeding appear to come *via* the cerebral ganglion neuron CBI-2 (Friedman et al., 2015). FCAP has not been implicated in feeding in any other species. The *M. leonina* SLB cells express peptides from the MIP-related prohormone, which is found in one bilaterally paired set of buccal neurons in *A. californica* (Fujisawa et al., 1999), and many small buccal neurons in *L. stagnalis* and *Helix pomatia* (Elekes et al., 2000). In each species, application of MIP-related peptides drives contractions of the gut. Finally, the *P. californica* VWC contains peptides from the QNFLa prohormone that is a homolog to the *A. californica* pedal peptide 4 prohormone (Green et al., 2018). Pedal peptide 4 has not been investigated physiologically, but in *Biomphalaria glabrata* was observed to be less abundant 12 days post-infection with the parasite *Schistosoma* (Wang et al., 2017).

What are the implications of peptide co-localization in these neurons? Co-localization suggests co-transmission, which can increase the flexibility of post-synaptic control. Co-transmitters, particularly those released from different prohormones, can confer numerous possible abilities onto a single neuron, notably, the modulation of a different neurotransmitter’s effects (Kiss, 2011), more refined control of a single target (Brezina et al., 1995; Vilim et al., 2010), or the differential control of multiple targets (Svensson et al., 2019). This final mechanism seems especially possible in the VWC/SLB cells, which affect both feeding circuitry and the gut. Interestingly, SCP and FMRF co-localize in a cerebral interneuron in five different nudipleurans, including *H. crassicornis*, *M. leonina*, and *P. californica* (Lillvis et al., 2012). We cannot say definitively what each peptide does in these three cells, but it seems possible that SCP is released to drive esophageal contractions, and the other peptides to regulate feeding circuits.

Additionally, what are the functional consequences of the unique aspects of each neurons’ peptide profiles? Differences in in the intrinsic properties and synaptic wiring of homologous neurons can lead to subtle differences in behavior (Newcomb et al., 2012; Ding et al., 2019), and it may be that these chemical differences are another mechanism of this change. *M. leonina* differs markedly from the other species in this study in the lack of a buccal mass, and differs further in its prey capture apparatus, feeding mechanics, feeding bout duration, and prey. *P. californica* and *H. crassicornis* differ from each other in prey choice and the relative size of their feeding apparatuses. Additionally, the *M. leonina* buccal ganglion is considerably smaller than that of the others, consisting of only 30 to 40 neurons (Trimarchi and Watson, 1992). The differences in peptide profiles may relate in part to these anatomical and behavioral differences.

Finally, in prior studies, neuron homology has been inferred based on synaptic wiring, neuroanatomical position, function, and overlap in immunohistochemical staining (Faulkes, 2008; Lillvis et al., 2012; Sakurai and Katz, 2019). The data in this study do not address the first three characteristics, but do suggest that limits should be placed on interpretations made based on immunohistochemical staining. Our data suggest that at least some of the peptides expressed in homologous neurons will not overlap, and thus if staining is performed for a peptide found in only some of the neurons, it may lead to incorrect conclusions regarding homology.

## Conclusion

Characterization of the neuropeptides present in a variety of animals is essential to our understanding of neurotransmission. Combining *de novo* transcriptomics and peptidomics allows us to examine the functional consequences of different peptide profiles without requiring a genomic assembly. The usage of different species in this work helped reveal what is “typical” of neuropeptide signaling, which is essential to the translatability of comparative research. Examining neuropeptides in nudipleuran sea slugs furthers this goal, and moreover, does so in a clade that has provided great insight into neuronal circuits.

## Data Availability Statement

The original contributions presented in this study are included in the article/Supplementary Material. Further inquiries can be directed to the corresponding author.

## Author Contributions

CL – conceptualization, methodology, data collection, and writing. ER – conceptualization, methodology, and writing. BS – methodology, data collection, and writing. RG and JS – conceptualization and writing. All authors contributed to the article and approved the submitted version.

## Funding

The project described was supported by Award Number P30DA018310 from the National Institute on Drug Abuse (NIDA) and the Friday Harbor Labs Research Fellowship Endowment. The content is solely the responsibility of the authors and does not necessarily represent the official views of the funding agencies.

## Conflict of Interest

The authors declare that the research was conducted in the absence of any commercial or financial relationships that could be construed as a potential conflict of interest.

## Publisher’s Note

All claims expressed in this article are solely those of the authors and do not necessarily represent those of their affiliated organizations, or those of the publisher, the editors and the reviewers. Any product that may be evaluated in this article, or claim that may be made by its manufacturer, is not guaranteed or endorsed by the publisher.

## Supplementary Material

The Supplementary Material for this article can be found online at: https://www.frontiersin.org/articles/10.3389/fphys. 2021.809529/full#supplementary-material

## Abbreviations

ARC: accessory radula closer
CNS: central nervous system
FMRFa: FMRFamide
LFRFa: LFRFamide
MALDI: matrix-assisted laser desorption/ionization
MIP: myoinhibitory peptide
MS: mass spectrometry
SCP: small cardioactive peptide
SLB: small cardioactive peptide B large buccal
VWC: ventral white cell
TOF: time-of-flight
