# Supplementary material for: Comparative Analysis of Neuropeptides in Homologous Interneurons and Prohormone Annotation in Nudipleuran Sea Slugs
Source: Front Physiol. 2021 Dec 23;12:809529. doi: 10.3389/fphys.2021.809529 (PMC8735849; doi:10.3389/fphys.2021.809529)
Supplement: Supplementary file 1 [file Data_Sheet_1.PDF]

Supplementary Material

```
Lymnaea         MEHI-----SLAMVLAFLTALICTAHSDDGAHSSSVKAFESSNHESHQPQKRSPSASPSA    55
Melibe          MATTS-VSLSIMVLFLYVATALSQETAE-LSQ----RGEMQQPTSRHQAQKRST--LIEP    52
Hermissenda     MATVSTSCLWLVVLCVWMGPVISSESSD-ITQ----SDLVAGSPAHHQAQKRSAAALLEQ    55
                *         : ::  ::   :.   ::  ::              ** ****     .

Lymnaea         YDE---------DLLLADSGLMDAPYN-------------LDDVDKRNTLFRFGKRQ-GS    92
Melibe          QEPGYIGEEQESEG-----PVYINPYD---------------DLSKRSSLFRFGKR--NS    90
Hermissenda     VDSAYPDVDMDQTYPDASSFLGGDPYDDELLNTNNNNKRTLFRFGKRGSIFRFGKRARGS    115
                 :                  :   **:                ..**.::******  .*

Lymnaea         LFRFGKRGGSLFRFGKRGTLLRFGKRGGSLFRFGRSGPSDSSDSE--NMEDKRTLFRFGK    150
Melibe          LFRFGKRGGSLF---------RFGKRGGTLFRFGRSGPQS------NEGMKRNSLFRFGK    135
Hermissenda     LFRFGKRGGSLF---------RFGKRGGSLFRFGKRSDDQDWNENGAASDKRASLFRFGK    166
                ************         *******:*****: . ..          .: :******

Lymnaea         RSDLD-LLRDALAEAEYSNLPVYAENE--PMKRDIQGFHWGMDNDEQ        194
Melibe          RADDS-QVLDAL--ESYYGLPLAAQLEEVQRKRNVNSFHWGDSE---        176
Hermissenda     RAHDSDNVLDAL--ESYYGYPSYALSDDVDSKRNVNSFHWGESE---        208
                *:. .  : ***   .* . *  *  :    **:::.**** .:
```

Figure S1: Multiple sequence alignment for the LFRF prohormone, showing the previously described prohormone from *L. stagnalis*, and the newly characterized prohormones from *H. crassicornis* and *M. leonina*. Highlighted stretches indicate peptides present in the VWCs; underlined blue stretches indicate peptides previously characterized in *L. stagnalis*.

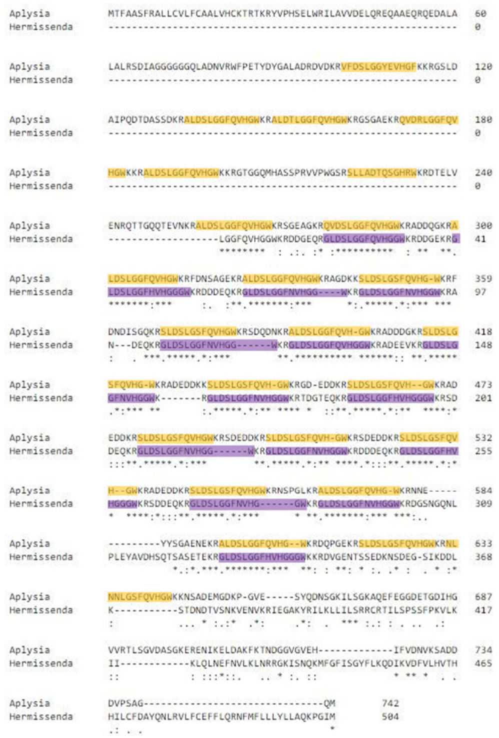

Figure S2: Multiple sequence alignment of the *A californica* FCAP prohormone to a partial protein in *H crassicornis*. Yellow lines indicate previously identified peptides in *A. californica*, and purple bars indicate the peptides found to be present in the *M. leonina* SLB cells. The protein is incomplete, lacking a signal sequence and seeming to lack several peptides found on the *A. californica* prohormone, but nonetheless shows similarity to the *A. californica* prohormone; 15 peptides (three types, with multiple copies of each) were encoded in the region of the *H. crassicornis* prohormone that was available to us, and each of them aligned to an *A. californica* peptide. It will be of interest to determine if the complete *H. crassicornis* prohormone produces variants of the FCAPs other than the three found here. The *A. californica* prohormone produces eight FCAP variants, but only four in the region of the prohormone that aligns to the partial *H. crassicornis* prohormone, and thus, it is possible that those from *H. crassicornis* have a similar pattern.

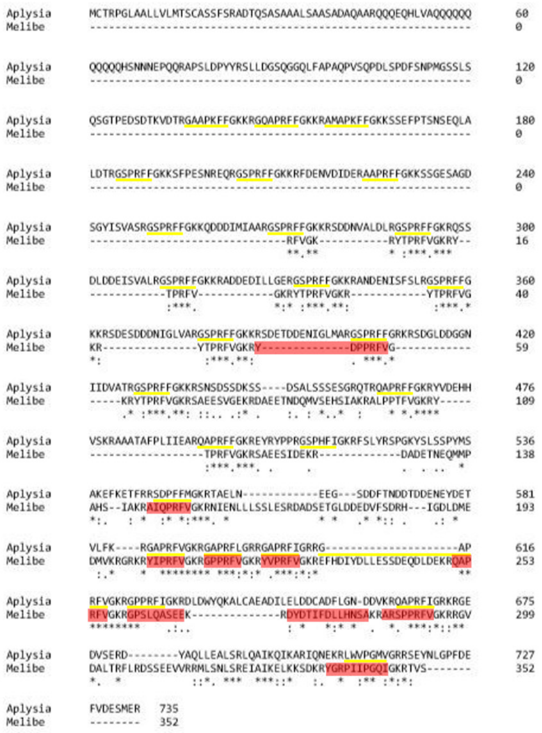

Figure S3: Multiple sequence alignment of the *A californica* MIP-related peptides prohormone (Accession no: NP_001191614.1) to a partial stretch of a protein in *M. leonina.* Yellow lines indicate previously identified peptides in *A. californica*, and red bars indicate the peptides found to be present in the *M. leonina* SLB cells.

```
Aplysia_californica             -MRPWCQLALLACLSLKWLTSHVTAESFLCDDSELCEN-GYLRFGRSMSVEEPHF--------RL------ERRSYPPVVYHKRFLRFGRSQEPDIED--YARAIALIESEEPLYRKRRS   102
Pleurobranchaea_californica     -MTSLCCLALTALLALQVSSHNARAQSVICDDPELCLPKRFLRFGRSGSESSDGYYLTPHLAFSSGRRFVRLGRAYEPY-QDKRFLRFGRASAGGQRSEESLLREALMQAEEPLYRKRRS   118
Hermissenda_crassicornis        MKLTWSPPTIVVLLMVQWSSVMSQTRRRISCDEISCPSKRFMRFGRDM-GP-DPY-------YRLRRQFFRLNRSYQPY-QDKRYLRFGRSQLGDL---QGFDDNYNTDIVEPYSRKRRS   107
Melibe_leonina                  MKSWMSPLAIVALMAVHWATFLVQAESIICDNPELCLSKRFLRFGRGPRAE-DPF-------YRISRQFVRFGRAYRPY-HDKRYLRFGRSQQPNV---DDIYNKALLQLEEPYSRKRRS   108
                                     .  :: . : ::  :    :.  :. :   *    ::****.       :                 *:* *   .**:*****:.  .           :  **  *****

Aplysia_californica             ADADGQSEKVLHR-------ARREAESEHKSLEEVSPDTKQDVEKRDADDVLDAEKRFMRFGKRFMRFGRGSSDDDESGDDDVQDLTDIGDGLGGEGEVNKRFMRFGKRFMRFGKREDGE   215
Pleurobranchaea_californica     AQQDEADEAEEGAAEGAQQRMKRSPAEEEEK----SEGAIKALQKRDVGGG--------------------SA-AGDAEEEDIISRQILGLGGGQVGESGDVIDGFRKRFMRFGRVPG--   211
Hermissenda_crassicornis        LDTDSQLEIT-------HNHVRRSVDQSELEQPG-NDDTSKMIKKRAIAEK--------------------R-------------------------------------FMRFGRDFSRD   162
Melibe_leonina                  PDADSQLKLT-------HNQFKRSPDQSEYHEGLVSDDVSNAIKKRSVDDD--------------------DM---STRSGDVIDR----------------------DLMGLGPATG--   174
                                 : *   :                :*.  ...      . .. : ::**                                                            :* :*   .

Aplysia_californica             PDKRFMRFGKSMADNDLDKRFMRFGKRFMRFGKSLPDSEVDKRFMRFGKSVDGDVDKRFMRFGKSVDG-----DVDKRFMRFGKSVDGDVDKRFMRFGKSVDGDVDKRFMRFGKSV---D   327
Pleurobranchaea_californica     -----------DEHEEAEKRFMRFGKRFMRFGRQ-----AGKRFMRFGRGLE--SAKRFMRFGRPSNAALEGLEGEKRFMRFGKRDTKAVESRTGADSQ---------------------   292
Hermissenda_crassicornis        EDK----DDINEENSQFSKRFMRFGKR----------------FMRFGRNHA--VGSTGD------ILAAPLSETNKRFMRFGKRESIDDANV-ENVLEEANINKLNNVLSLASQLKSQQ   253
Melibe_leonina                  --D----GGTGNAHNDLDKRFVRFGKRLIP---A-----AAKRFMRFGRNDN--M------------------LGASKRFMRFGKRKMGEEEHGEPRDLSREELNEIHDVLSLADQLKSQT   261
                                             ..: .***:*****                 *****:.                    .********                .

Aplysia_californica             GD-VDKRFM--------RFGKSVDDDVDKRFMRFGKSVDGDVDKRFMRFGKSVDDAVDKRFMRFGKSVDSDLDKRFMRFGKSVGSDEVDKRFMRFGKSVGSDEVDKRFMRFGKSLGTDDV   438
Pleurobranchaea_californica     ------------------------------------------------------------------------------------------------------------------------   292
Hermissenda_crassicornis        RSLVDQSELEQPESDSTTKNIKKRAILEKRFMRFGRGFSRDDDEDNID------------------DENSQFSKRFMRFGKRLVP---VKRFMRFGRNLAGDILVA---------PLSET   343
Melibe_leonina                  G--------------------QKNELVDKRFMRFGKREDGTERA----------------------------------------------------------------------------   285

Aplysia_californica             NKRFMRFGKSLGTDDVNKRFMRFGKSLGTEDVD--KRFMRFGKSLGTDDVDKRFMRFGKSLGTDDVDKRFMRFGKSLGTEDVDKRFMRFGKSLGTDDVDKRFMRFGKSLGTEDVDKRFMR   556
Pleurobranchaea_californica     ------------------------------------------------------------------------------------------------------------------------   292
Hermissenda_crassicornis        SKRFMRFGKRKNLDDSNIEDVESN--LEEANMNSLNNVLSLANRLK---------SEAGELG--------------GGLYGQEKRFMRFG------------------------------   408
Melibe_leonina                  ------------------------------------------------------------------------------------------------------------------------   285

Aplysia_californica             FGKRFMRFGRSVGDSKYRSASSESVMTTDSKQTTEQATNKS      597
Pleurobranchaea_californica     -----------------------------------------      292
Hermissenda_crassicornis        -----------------------------------------      408
Melibe_leonina                  -----------------------------------------      285
```

Figure S4: Multiple sequence alignment for the FMRF prohormone. The previously described FMRF prohormones from *A. californica* and *P. californica* aligned to newly characterized prohormones from *M. leonina* and *H. crassicornis*. Yellow lines indicate peptides previously characterized in *A. californica*; highlighted stretches indicate peptides present in the VWC/SLB cells.

Table S1: Predicted prohormones from *H. crassicornis.*

>Abdominal_ganglion_neuropeptide_L11_H_crassicornis

MPTQITLHLLSLLVVLLCLVAMVAPRRINCRRYPFHNACRGISAKRSFSPSSSSSSSSSSTAPR
LNLLNRLMLLSHNAEDRIDDQGMDKDAEGLPSSLGLRLNMQQPPQHHLQQQQQKQRQYR
QQPFDGDSERRGEDVDDADEAVNRLRQHALHTIQLD

>Abdominal_ganglion_neuropeptide_L5-L67_H_crassicornis

MKTTEVLVCGSCMVLALILCEASPAWRPQGRFGKRTHVQAIPRVIETGVSEDGISNLMEIPIE
LLYTHSDLSQLKTKPWLCSNSRISGYPPCGGTKKGSTSSSLSLASTSRMKNLNALADYLNL

>Abdominal_ganglion_neuropeptide_R3-14_H_crassicornis

MDLLRVCIVLTLCVAMMTQAVLSAPAFGQDLDTIDDSQLEMDPELAVFRERRDLADVDDES
PELLSRLRRQVAQMDNGRRRYGSHGHRRRGRFHSRRLYQSRRNYRARGRVTDW

>Achatin_H_crassicornis

MTSSISLLILAFLATLLTFDLVTASGLKFPESGEYLIDDVDNYPQDEFLGRLESAEDGGKRGFA
DKRGFADKRGFADKRGFADKRGFADKRGFADKRDLLLKLLRQRYQGSPLQGRNAFSRLLV
KHGILYN

>AKH_H_crassicornis

MHPHQHQQTVHQHMSLSVLLLLVLCVCSGLAQIHFSPGWEPGKRAMDDSEIRSNKLACYD
QFDMTLLMDILKLVKRQAEKLSYCTKGCPQL

>Atrial_gland_and_califin_peptides_H_crassicornis

MVMESRRKEAEALRKKLLEIGKRSESESDLEPESSGDDTSSIMNKRQRYLSVNQPLHILTSM
VMESRRKEAEALRKKLLEIGKRSESESDLEPE

>Buccalin_H_crassicornis

MASPTQVLTLKLFLLFVLHQVILGEKDTNSNEKETEWVQKRQVDPFSFASGIGKRGVDPFSF
ASGIGKRGMDPYSFAGGIGKRGMDPFSFASGIGKRGMDPYSFAGGIGKRGMDPFSFASGIGK
RGMDPYSFTSGIGKRGMDPYSFTSGIGKRGMDPFSFTSGIGKRGMDPFSFAGGIGKRGMDPF
SFTSGIGKRGMDPYSFAGGIGKRGMDPYSFASGIGKRGAESFSSDDSTSKHSDSTRRRRHAM
TSQGNSEVTLSDLLKSPHQPLGWTHNVGLATTPSGCERGTIPDYDTTRGQTHSTRTSTLNYD
VITTTTSTTTNHAVNKEL

>Cerebral_Peptide_H_crassicornis

MLAQIVLLISIAVIDLTQSSDSAAAFNSAKKVTSSENSLSPNRHKRRVTDEALFGGDGDTQQA
VEALKRAPGWGKRFFDGMAEAEKRDPGWGKRSSDFDDEEDQDSNDKRAPGWGKRAPGW
GKRSPGWGKRGWGKRAPGWGKRAPGWGKRSDDSDLCQRLDTIADSFLMEARKVNAIFLK
ECGSLETGNDPFRK

>Cerebrin_H_crassicornis

MPSRLSSLLCLLTILVLAILVQASSGSSYNAYKSQTLDDNSKREAMSLLTRLADIIQYGNART
HYIKRNGGTSDTLYNLPDLMELGKRR

>Enterin_H_crassicornis

FVGKRAPEFGHGFVGKRAPEFGHGFVGKRGLEFGHGFVGKRAPEFGHSFVGKRAPEFGHGF
VGKRAPQFGHSFVGKRAPQFGHGFVGKRAPQFGHGFVGKRAPQFGHSFVGKRAPQFGHSF

VGKRTDYDAKILSILDNAITDTTNEELSTNGFDELKSQASTNPEINQSYFNANNKDIDYGFEV
ANEKETDSDDSSKFENSSNEVVLVPGEESSS

>Enticin_H_crassicornis

NDKITARLRRCDDDRQVRAKQNTLPIHSLLLVP

>FCAP_H_crassicornis

MKGGAFVPVFVLGVLCVLGVRAQNTLTASDLHDLETLGRIVESDIRRELSQTRRNVLNNLL
GASLKQHHVASASDDQPTDFGTDSLYSNIIDLELANNDHGQTQHRGLQKRYLRSFPFNYRRS
FDSLGGMQVHGWKKRSAETVPEYHFSSRAKRSADKEPEAVAKESSSQESQERVARGLDSLG
GFNVHGGWKRGLDSLGGFNVHGGWKRDDGEKRGLDSLGGFQVHGGWKRDDGEKRGLGG
FQVHGGWKRDDGEQRGLDSLGGFQVHGGWKRDDGEKRGLDSLGGFHVHGGGWKRDDDE
QKRGLDSLGGFNVHGGWKRGLDSLGGFNVHGGWKRANDEQKRGLDSLGGFNVHGGWKR
GLDSLGGFQVHGGWKRADEEVKRGLDSLGGFNVHGGWKRGLDSLGGFNVHGGWKRTDG
TEQKRGLDSLGGFHVHGGGWKRSDDEQKRGLDSLGGFNVHGGWKRGLDSLGGFNVHGG
WKRDDDEQKRGLDSLGGFHVHGGGWKRSDDEQKRGLDSLGGFNVHGGWKRGLDSLGGF
NVHGGWKRDGSNGQNLPLEYAVDHSQTSASETEKRGLDSLGGFHVHGGGWKKRDVGENT
SSEDKNSDEGSIKDDLKSTDNDTVSNKVENVKRIEGAKYRILKLLILSRRCRTILSPSSFPKVL
KIIKLQLNEFNVLKLNRRGKISNQKMFGFISGYFLKQDIKVDFVLHVTHHILCFDAYQNLRVL
FCEFFLQRNFMFLLLYLLAQKPGIM

>FMRFa_H_crassicornis

MKLTWSPPTIVVLLMVQWSSVMSQTRRRISCDEISCPSKRFMRFGRDMGPDPYYRLRRQFFR
LNRSYQPYQDKRYLRFGRSQLGDLQGFDDNYNTDIVEPYSRKRRSLDTDSQLEITHNHVRRS
VDQSELEQPGNDDTSKMIKKRAIAEKRFMRFGRDFSRDEDKDDINEENSQFSKRFMRFGKRF
MRFGRNHAVGSTGDILAAPLSETNKRFMRFGKRESIDDANVENVLEEANINKLNNVLSLASQ
LKSQQRSLVDQSELEQPESDSTTKNIKKRAILEKRFMRFGRGFSRDDDEDNIDDENSQFSKRF
MRFGKRLVPVKRFMRFGRNLAGDILVAPLSETSKRFMRFGKRKNLDDSNIEDVESNLEEAN
MNSLNNVLSLANRLKSEAGELGGGLYGQEKRFMRFG

>FMRFa-related_neuropeptides_H_crassicornis

MVQWSSVMGQTRRRISCDEISCQELPGTDLWESCCNQKTEVLDVSFDEKRSDSKNDVALEK
ESFLTNLKRQDPFLRFGKRQDPFLRFGKRQDPFLRFGKRQDPFLRFGKRQDPFLRFGKRQDPF
LRFGKRQDPFLRFGKREGDGPPPKFMRFGR

>GNRH_H_crassicornis

MTSVARLAICLVVLLAMLEATNAQNVHFSNGWYPGKKRSSPSLSSAAAAAAAAAAAANPL
LSGASSSSGSPSAFSACSVRPDLGDIISKLIMEEIVRLHSACQVGVPRDLRDILETAASKQTKW

>Insulin_H_crassicornis

MVPHPNFSPMCLLALTVTLMYTLPAPAAANYEHYCDSATFRIQRNGQCGESLANTILSLCRA
LSNSFVGKYMMKKRTPAKGAVQKRLSDISLTKSDAFSYLAKRQSSQNVVCECCVNRCGITE
MFQYCELPPLTFKRRIRGDDDNSSPLQKDRRHHSNLEDSDYKGST

>LFRFa_H_crassicornis

MATVSTSCLWLVVLCVWMGPVISSESSDITQSDLVAGSPAHHQAQKRSAAALLEQVDSAYP
DVDMDQTYPDASSFLGGDPYDDELLNTNNNNKRTLFRFGKRGSIFRFGKRARGSLFRFGKR

GGSLFRFGKRGGSLFRFGKRSDDQDWNENGAASDKRASLFRFGKRAHDSDNVLDALESYY
GYPSYALSDDVDSKRNVNSFHWGESE

>MIPR_H_crassicornis

GKRYTPRFVGKRSAEESVGEKRDAEETNDQMVSEHSIAKRALPPTFVGKRYTPRFVGKRSA
EESIDEKRDADETNEQMMPAHSIAKRAIQPRFVGKRNIENLLLSSLESRDADSETGLDDEDVF
SDRHIGDLDMEDMVKRGRKRYIPRFVGKRGPPRFVGKRYVPRFVGKREFHDIYDLLESSDE
QDLDEKRQAPRFVGKRGPSLQASEEKRDYDTIFDLLHNSAKRARSPPRFVGKRRGVDALTRF
LRDSSEEVVRRMLSNLSREIAIKEL

>Myomodulin1_H_crassicornis

MHIYGLLLLLTLSAFHSSSDAAEENLASNNLESQHNVVAPSRVKRQGMNILRLSRTASAGGF
PNDYPQYQDQDRDDVLALEKISEELQSPYLNEISPSDYYQALNSLQDSYSHSRYRRSTPADD
MMVTPVPLVDDSGADGLKIDENDDDSYFLYQDLADDDSVVVDDDGKAWDVKTGEELSKR
GLNMLRLGKRDNRNTKRLNMLRLGKRESDNEKRNMNTLRLGKRQMESFDSEFGAPSDLSS
LGHNTKRFPMLRLGKRESDNEKRNMNTLRLGKRQMESFNSEFGAPSDLSSLGQDSKRFPML
RVGKRESEDEKRNLNMLRLGKRQMNMLRLGKRQMNMLRLGKRQMNMLRLGKRQLNML
RLGKRQLNMLRLGKREDADDNTAAKRNLQMLRLGKRQEDDVEKRNLHMLRLGKRSVENA
PVNKAE

>Myomodulin2_H_crassicornis

MFKMLTNCPWYCCAVTFALYLTASAIPVVSSEDVDSKRPWDMVRVARGLQMLRLGKRDS
NEAKEPITDEEFRQLLDFLSESRDPTRRQPPLPRYGRELVTRRELLDFLERVPTPTTYMRPPPR
AGRYKRSVVVDENPFSRIRSVVRPRIGRYLQPVHFKHLQAKAISRPRIGRDQLYQAAAAAAA
AAAGAGNGPAVNGDDGGIMDIYQ

>NdWFa_H_crassicornis

MAKIFAAICVVVLVLNVISSSPAQANWFGKRGDQEDILGYILRQSDSHQDSALSAESAMAAI
ERIVKNWKLARKVGIATSTQK

>Neuroactive_Polyprotein_R15_H_crassicornis

MVTILLGSHISFTSARHTEKRDADPRDDQRIGTGGLEMMCKLHPRNCPGGLLLKREESLINRL
RRIFPNDVYSGTNPNANPTNNNERSETAFVLEDDDDEIPSGSIDILPNSLSSSASASSLNDLGN
YPAAPVERRDSRDVVERPMARLLD

>NPY_H_crassicornis

MHKFLLVALLIASLATMGVYSMETMLAPPARPREFKSPNELRRYLKALNEYYAIVGRPRFG
KRTNNELRTSDFFRNRGDDNDASESMGWSDY

>Neuropeptides_CP2_H_crassicornis

MEFSHVTSVFLITMAMTLAVSCALPYQTLFDPDTAELADSVVERRPASPAEYGNEELDRLRR
LVALKLMRRELISDDAQNNKDSRIAKRLDFGFAGLDNIDHIITTLDRKEAMKSMEKPRLLQN
MQRHG

>PEP1_H_crassicornis

MNTLRIILASLLVISAFAADPEKTDEDIEAQPVKRSTDDDEEFEKKSFDPISGAHGLNGFIKRSF
DPISGSYGLQGFAKRSFDPISGAHGLNGFVKKSFDPISGSYGLQGFAKRSFDPISGSYGLQGFA
KRSFDPISGAHGLNGFVKKSFDPISGSYGLQGFAKRSFDPISGAHGLNGFVKKSFDPISGAHG
LNGFVKKSFDPISGAHGLNGFIKRSFDPISGSYGLQGFAKRSFDPISGAHGLNGFVKRSFDPIS

GAHGLNGFVKKSFDPISGAHGLNGFVKKSFDPISGAHGLNGFVKKSFDPISGAHGLNGFVKK
SFDPISGAHGLNGFVKKSFDPISGAHGLNGFVKKSFDPISGAHGLNGFVKKSFDPISGAHGLN
GFVKKSFDPISGAHGLNGFVKKSFDPISGAHGLNGFV

>PEP2_H_crassicornis

MTSSTTRFAFLLVFAVTVAVGSEVVDGEIQESPAGSREKRSIDSIGSFFIKRDTRQASLNKARN
DASAWFHHDKRGIDSIGSHYVKRHVDSIGSTFVKKSVDSIGSTFIKKNVDSIGSTFVKKDDND
NFEKRAIDSLGSSFIKKRHIDSLGSSFIKKRHIDSLGSSFIKKRPIDSLGSSFIKKRPIDSLGSSFIK
KSVDSIGSSFVKRPIDS

>PEP3_H_crassicornis

MGVLTIAGILLFSHALHQTSAAPGHSPHQIDSDKLVSSVHHPLDSDAGTPQSNDQLTTSNVEP
VSRGSPISRQVDAKSDIPETSDTYLSAPEAETVVSDGEKETREQIVKDSSSSSDIEKRSFDPISG
NSAFSGFGKRTFDPISGNSAFTGFGKRNFDSISGNSAFSGFGKRTFDPISGHSAFSGFGKRTFD
PISGNSAFSGFGKRTFDPISGNSAFTGFGKRNFDSISGNSAFSGFGKRTFDPISGNSAFSGFGKR
TFDPISGNSAFTGFGKRTFDPISGNSAFTGFGKKRSFDSISGNSAFSGFGKRTFDPISGNSAFSG
F

>PEP4_H_crassicornis

LSTSNSFSGMNQNFLNPMATRADNNEEILKEQLYYRLRDILRRVKSSRQKKSESVKEKEDGP
IIDWSRSDASSGDLERLEELSPESLDLLDMDDLNKLNMEDGFPSFAKKFDVLTDSGMSGMN
QNFLQKKSEIADMDLFSDPEDYPVKRFNTLSSSDLAGFSQNHFKKRFNQLSSSGLSGFEQNH
LKKRFLSAASPMGGLGQNHIKRNIFLSQRTPMGNIGQNHVKRFDPLASSGMSGFDQNHLKK
RFNSLSSSGLSSFDQNHLRKRQNALDGFNADQTMNGFNLLSRSGMSGFDQNHLKKRFDSLS
SSSMSGFDQNHLKKRFNALTSSGMSGFNQNYLKKRFLSAASPMGGLGQNYVKREFSMSQR
TPMGGFGQNHIKRFDPLSSSGLSGFDQNHLKKRFNSLTSSGLSSFDQNHIKRNFDTLSSSGLS
GFNQNHIKRFDPLS

>Pleurin_H_crassicornis

MYQVLQLVTLSLLAGSTCAVFYIPGSVVSYPKMGKRAFYTNATGNRYPLMGRRTSPTNDQG
QGQSQGQNAEMAAVAAGALVQNGDEFNKRGVFTKGPHRSFPRVGRSDASSDSFDATVSGK
IRSQILKGLAERLDRNILESSEISNLVAAGQQQGAQSAESSESLKALQLPHMLFLVFDENGDN
QLSKAEFTLGLAKALEQNLLC

>PRQFV_H_crassicornis

SLGEEVKRPAFSSWSGKRSLGEEVKRPAFSSWSGKRSLGDEVKRPAFSSWSGKRSLGDEVK
RPAFSSWSGKRLAHSYLIAPKRPAFSSWSGKRSFLDNAVPSKRPAFSSWSGKRSLDTEDMLS
KRPAFSSWSGKRSVDEESLLNAKRPAFSSWSGKRSAELIDSKMLSKRPAFSSWSGKRSSDEK
EMSKRPAFSSWSGKRSLDTEGLLSKRPAFSSWSGKRSSDGEGLLSKRPAFSSWSGKRSSDDE
GILSKRPAFSSWSGKRSSDDEGILSKRPAFSSWSGKRSSDDTILASKRPAFSSWSGKRSAERID
NNMLSKRPAFSSWSGKRSSDGEGVLSKRPAFSSWSGKRSHGDDIITLKRPAFSSWSGKRSLN
DDEISKRPAFSSWSGKRSIDDNEMSKRPAFSSWSGKRSVDENEMFKRPAFSSWSGKRSSDDG
DDISKRPAFSSWSGKRSVDDTMLASKRPAFSSWSGKRSVDANEMSKRPAFSSWSGKRFAHD
DDISKRPAFSSWSGKRSSDGDDISKRPAFSSWSGKRSSNENDMSKRPAFSSWSGKRSGNDNE
MSKRPAFSSWSGKRSVDENDMSKRPAFSSWSGKRSSDDGDDISKRLAFSSWSGKRSVDDTM
LASKRPAFSSWSGKRSVDENEMSKRPAFSSWSGKRSVDDTMLASKRPAFSSWSGKRSGNDN
EMSKRPAFSSWSGKRSLGEEISKRPAFSSWSGKRAYHGDLFNIFNPLDFAGFYQNNFSKNDD
KSKINSIKRPSFSAWSGKRSL

>SCP_H_crassicornis

MELTMPKATLSLTLLFVVICTVDAVNYLAFPRMGRSGYLAFPRMGRAQAKALTASEQDGEC
CGIGLKSEFAIAEDGKEEMRNVCTASISVCCEGLREVADEKPDGTVYSMCVPDVMKLYPSSL
TKLKTLLSK

>Sensorin_A_H_crassicornis

MSSSFSWKSLLQTCLVLVLVICILVPDDVMAIEKRKSRSNRRQYRTVRYRVGYIYGKRSKPM
EGENASSKLFDAISRRLIPLPELIDMLRENPEILMQVVRHLDKDDDGYITVADLM

>Temptin_H_crassicornis

MVSFQLLLALASFLAAAVIVVPCQGYPFFAKMIPNAYRVKHPCLFFFPWLGVGHVNAFGGIA
LNAFGEDFQAEGLKWTKDLCEMDSDGDGRSNGQELGDPDCEWSPGKTPKRQVFITHPGYK
RFDPEG

>Whitnin_H_crassicornis

MENHQSILLVILGSLLCASLVSCLPKRSAEDILQDTSGMVLDKRPKYMDTRRDLDVFKDLVL
MSLQELVDEGRLEPSTLPEEDRETSKPVEKRRYMGLCMHRQANQYIPFPCLRTGR

Table S2: Predicted prohormones from *M. leonina.*

>Abdominal_ganglion_neuropeptide_L11_M_leonina

MIATPYRDFKKWSRIDCTRYVFHPVCRGVAAKRSSIPSLSSSSSSSSLSSASSFPSKLGALENL
AKIVAVENAKFKEKCLTSRFVAANSTANRTQKMRIRTHFRTSYACTHF

>Abdominal_ganglion_neuropeptides_R3-14_M_leonina

MDLLRVCIVLTLCVAMMTQAVLSAPAFGQDLDTIDDSQLEMDPELAVFRERRDLADVDDES
PELLSRLRRQVAQMDNGRRRYGSHGHRRRGRFHSRRLYQSRRNYRARGRVTDW

>Abdominal_ganglion_neuropeptides_L5-67_M_leonina

MKTTEVLVCGSCMVLALILCEASPAWRPQGRFGKRTHVQAIPRVIETGVSEDGISNLMEIPIE
LLYTHSDLSQLKTKPWLCSNSRISGYPPCGGTKKGSTSSSLSLASTSRMKNLNALADYLNL

>Achatin_M_leonina

ADKRGFADKRGFADKRGFADKRGFADKRGFADKRGFADKRGFADKRGFADKRGFADKRG
FADKRDFLLRMLRQRYQASPLQGRNNALSRLLVKHGILY

>AKH_M_leonina

MHRSSRTTLTFLLLVLCLCSSIAQIHFSPGWEPGKRSMEEPDRSTKLTCYDQLDMSLIMDIIRL
VRRQAEKLSYCMKGCPRLGRDM

>Atrial_Gland_and_Califin_Peptides_M_leonina

MPTSKPFLQNAAAIFVLLLLTAEVTNSIAAEGRPKVSTISEDRDVSDLRNAGNSPAGDRTPVF
PLTPETDSRRTSSGTGSSRLSSVGKELRVGVVRNGIFSECIQRQDGSFDTECRFFESAVNPSQG
FGEIDDSAGAGSGSIEPTRPNSDKRRLRFIKRRLRFVRRNDVGQDSALGKRRLRFIKKANFEE
ENSSPRGSEIRPEVISPPSRGSEASDSLSPPHAIRKRSVASAHDPAPMDLSSPIQGPSTTKRYLS
VNQALHILTSMVMESRRKEAEALRKKLLEIGKRSESESDLEPESSGDDTSS

>Buccalin_M_leonina

MALKLHLIISPLVLLLLSVACQITLGKDAKPKSKDWDKRDHDPFSFSAGIGKRGFDPYSFTAG
VGKRGFDPYSFTAGVGKRGFDPYSFAAGVGKRAFDPFSFTAGVGKRGFDPYSFAAGVGKRG
YDPYSFAAGVGKRGFDPFAFSAGVGKRGFDPYSFAAGVGKRGFDPFAFSAGVGKRDHHSKE
LSAKKRGLDPFSFSAGVGKRGFDPYSFAAGVGKRAHDPLKRAIDHLSFSSGIGKRRLEQYLL
QGGTKKRQLDPNYYRPAYGKKVRVVREEEEKPSQQERQPTGSCVGTSGLMSSTSI

>Cerebral_peptide_M_leonina

MLAQIVLLISIAVIDLTQSSDSAAAFNSAKKVTSSENSLSPNRHKRRVTDEALFGGDGDTQQA
VEALKRAPGWGKRFFDGMAEAEKRDPGWGKRSSDFDDEEDQDSNDKRAPGWGKRAPGW
GKRSPGWGKRGWGKRAPGWGKRAPGWGKRSDDSDLCQRLDTIADSFLMEARKVNAIFLK
ECGSLETGNDPFRK

>Cerebrin_M_leonina

MSQPNSWSLASLLSLLIISVLLVNQCHAASYAPSSYTATQLSDDSVRKEILSLLRRVATLVHY
THPGRQQFLKRNGGTADSLFNLPDLMDIGRRR

>ELH_M_leonina

MPTTKSTIHFLPLAIVLVVIATTTPSNAISRQSSLLAKLTSSTTTGPSNHEDGSLPSSSPRLLRAR
LDNPNWLMSCAGQIRLLRSQLLSQINNIDNDNNDNTNDNFPLGRLLSNNRNNIQVPRKFLVP
TDLCLNTAAKYLQQQQQQQHFAYEARVTLLHQRRANVNTNMNTDDTNMDAVAGSVGSD
RSSDDSRGEAFPADSYPSPNYSPSDYNNIYSSLVYRQAQNDDHDKTNSSPPPLQEIGGQSAYE
NNVNNVKERSSNADGEFGQGRRRLRLPGPGTAMAETTGENDGISDIAPAASSRVASTAATA
ATAPRQKRWQRLSVNQALMVLTDMVMHEQRQERLRKAEKMRAHLLSIG

>Enterin_M_leonina

MTMSCPTIRSLALLFCVFLAAIVVAESATKSNGRKASLNNLLNHIITLPFRRSPPKFNHEFIGK
RSTNSISGDSALLPGPRLLKLFPSSRQGRTNALNDFLSDIGVQPSLDLDTVEAEKRDAEIGEAD
GLVSDMIEAYGEKRGPPDFGHAFVGKRGPPDFGHAFVGKRGPPDFGHAFVGKRGPPDFGHA
FVGKRGPPDFGHAFVGKRGQPDFGHAFVGKRAPDFGHAFVGKRGPPDFGHAFVGKRTPDF
GHAFVGKRAPDFGHAFVGKRGPPDFGHAFVGKRAPDFGHAFVGKRGPPDFGHAFVGKRTP
DFGHAFVGKRAPDFGHAFVGKRGPPDFGHAFVGKRTLDFGHAFVGKRKPDFGHAFVGKRG
PPDFGHAFVGKRTPDFGHAFVGKRGPPDFGHAFVGKRTPDFGHAF

>FCAP_M_leonina

MTILPLSFLLALLSVQGMTSQLILSPSETKELESLVSLLSKGINQKLSPTRESVLNSLLRDSIQN
YYIKEQDDGQTEGEAPFNNLFDLKLASESLGTNGGTGIAKRFSNFFHKKGGFRRAFDSLGGM
QVHGWKKRSADFDSASNKPSRSRRSVGEQNDTSATNESNPGDSLSEGTKDLFSERGANVKA
KRALDSLGDFAVHGGWKRALDSLGGFNV

>FMRFa_M_leonina

MKSWMSPLAIVALMAVHWATFLVQAESIICDNPELCLSKRFLRFGRGPRAEDPFYRISRQFV
RFGRAYRPYHDKRYLRFGRSQQPNVDDIYNKALLQLEEPYSRKRRSPDADSQLKLTHNQFK
RSPDQSEYHEGLVSDDVSNAIKKRSVDDDDMSTRSGDVIDRDLMGLGPATGDGGTGNAHN
DLDKRFVRFGKRLIPAAAKRFMRFGRNDNMLGASKRFMRFGKRKMGEEEHGEPRDLSREE
LNEIHDVLSLADQLKSQTGQKNELVDKRFMRFGKREDGTERA

>FMRF-related_M_leonina

MKSWMSPLAIVALMAVHWATFLVQAESIICDNPELCRERLTVKSSDDNGHKGDKFDASNQ
HKEEKRSKYMRFGKREHEQLIRLQRRDAILDKYGGGDPMLRFGRQDPRFGGQDPILRFSRQT
VKASDDHGGTRGYLHMGKRDEKPIVRITKQDPMFDKYGGQDPMLRFGRDTQFMRIGKRDE
KPIVRITKQDPMFDKYGGQDPMLRFGRDTQFMRIGKRDEKPIVRITKQDPMFDKYGGQDPM
LRFGRQAVKASDDNGGTRGYLHMGKRDEKPTVRITKQDPMFDKYGGQDPMLRFGRHDPFL
RFGREDPFLRFGRQDPFLSSHPNGPVDQPSHLRYDRSAEVEDIEDLDARPHQVWKLFPLFGR
KRQLVNSPPESCSCSKTIIIVIMIVCYRQSIKYLYSCVGGGFELRFFFWFFFRNVIDKKINKLDI

>GNRH_M_leonina

MVKHAYTIPCSRTLKPSAGVRTSTSASASTTNGTARLAVFLVVILSLVEVNSGQNIHFSNGW
YPGKKRSSGLAVADAAASLAEASSPLAAASPGAAVPPSGARFSPSGARFLSSCMARPEVGEFI
NTLVMEELFRQPSSCVYSVSRGLRDMLESTARKLDKW

>Insulin_M_leonina

MSFSNVLPRHKSTYVFLVGLAALLISHAPSTWANYEHFCSSADFEYQRLGVCGDGLVNTISM
ACQFLNRNSRGKYLVKRSLPNEIDRQIRDLLVEKKDAFSFLSKRQSSQNIICECCVNSCNIME
LYQYCELPNLRSIRSENPKSNLQI

>LFRFa_M_leonina

MATTSVSLSIMVLFLYVATALSQETAELSQRGEMQQPTSRHQAQKRSTLIEPQEPGYIGEEQE
SEGPVYINPYDDLSKRSSLFRFGKRNSLFRFGKRGGSLFRFGKRGGTLFRFGRSGPQSNEGMK
RNSLFRFGKRADDSQVLDALESYYGLPLAAQLEEVQRKRNVNSFHWGDSE

>MIPR_M_leonina

RFVGKRYTPRFVGKRYTPRFVGKRYTPRFVGKRYTPRFVGKRYTPRFVGKRYDPPRFVGKR
YTPRFVGKRSAEESVGEKRDAEETNDQMVSEHSIAKRALPPTFVGKRYTPRFVGKRSAEESI
DEKRDADETNEQMMPAHSIAKRAIQPRFVGKRNIENLLLSSLESRDADSETGLDDEDVFSDR
HIGDLDMEDMVKRGRKRYIPRFVGKRGPPRFVGKRYVPRFVGKREFHDIYDLLESSDEQDL
DEKRQAPRFVGKRGPSLQASEEKRDYDTIFDLLHNSAKRARSPPRFVGKRRGVDALTRFLRD
SSEEVVRRMLSNLSREIAIKELKKSDKRYGRPIIPGQIGKRTVSLPPTFVGKRYTPRFVGKRSA
EESVGEKRDAEETNDQMVSEHSIAKRALPPTFVGKRYTPRFVGKRSAEESIDEKRDADETNE
QMMPAHSIAKRAIQPRFVGKRNIENLLLSSLESRDADSETGLDDEDVFSDRHIGDIDMEDMV
KRGRKRYIPRFVGKRGPPRFVGKRYVPRFVGKREFHDIYDLLESSDEQDLDEKRQAPRFVGK
RGPSLQASEEKRDYDTIFDLLHNSAKRARSPPRFVGKRRGVDALTRFLRDSSEEVVRRMLSN
LSREIAIKELKKSDKRYGRPIIPGQIGKRTVSQAPGLQSEDYDISIIQ

>Myomodulin1_M_leonina

MHIFALFLLTIISTGQLPCDADETAAITATSAPTTKTELLQKSSSPSSSSSFSSPSAPSRVKRGDE
DMMYRLTRGLRMLRLGKRDGGVFLPYDDSLYAPVVSDGDIYGAIGLRTRRSTPTDDTLVTP
ITPSDEGNEEEDGDNVDEIPAGLSERGEDEDTLFYPELRYIDDIEGSDGEGENGLDNGVETRD
LDLLPFGNPQQVLGSAGYDDVKRSLSMLRLGKRNKALISDNLDDNDNPLAMIGGPEFEAEK
RGLNMLRLGRSDRGWEKRGLNMLRLG

>Myomodulin2_M_leonina

MIAHCSTYYSFLTVFLFAAVYAEQMGSPGDLVVPKGSWDVMRIGRGLQMLRMGKRSSESS
KDGSSSSSGSGSPGVGGFSLSDGELQRLVEYVRGEMARDTTRRQPPLPRYGRDMISRREIMD
LLDSLSNRSYMRPAPRGGRYRRSTGGSRLGGPFSEDGNRFSRVRSVVKPRIGRFIQGLHLQTK
AISRPRIGRQPKIFSVGNDDQATGRMLEIY

>NdWF_M_leonina

MAKIFAAICVVVLVLNVISSSPAQANWFGKRGDQEDILGYILRQSDSHQDSALSAESAMAAI
ERIVKNWKLARKVGIATSTQK

>Neuroactive_Polyprotein_R15_M_leonina

MSVSNFRLSCLVTMVTILIGGHLSSTSARSTSRLTSPQDRLVRRDGYWARQGRIGTGGLALQ
CLSHPRNCPDGYRSPGHRAGKRGGDYLMELRQLFPRQQQASGVTSEEVPEEMQIQGAPRTG
NGVVREGDAEAIGHSGRTRAVSPQEQALVNSLLEVLMADQQLKRNSW

>NPY_M_leonina

MHKFLLVALLLASLATMGVFSEENMLAPPVRPKEFKSPNELRQYLKALNEYYAIVGRPRFG
KRTNSLRTSEYFRPRGDDADSVDSVGWSEY

>Neuropeptides_CP2_M_leonina

MELPCLTIALLLSVAMTLTVTSALPYPAWFQPQNKEDSYPVDLHQAVNTLQGMLARQLLKE
KFGPTLQNLAADNPPEEGSSISKRLDFGFAGIDNMDHILSVLARKQSSRGSGSGSAFFKNMKS
QG

>PEP1_M_leonina

MNHLRALVSCLLILYASAESEKVHASIADSEPVKRSADAERSGDEFAKRPFDQISGAHGLSGF
VKRPFDQISGAHGLSGFVKRPFDQISGANGLSGFVKRPFDQISGAHGLSGFVKRPFDQISGAH
GLSGFVKRPFDQISGAHGLSGFVKRPFDQISGAHGLSGFVKRPFDEISGAHGLSGFVKRPFDQI
SGAHGLSGFVKRPFDQISGANGLSGFVKRPFDQISGAHGLSGFVKRPFDQISGAHGLSGFVKR
QFDEISGTHGLMGFVKREAEEEEEDNSE

>PEP2_M_leonina

MDSIGSSFIKKNMDSIGSSFIKKNMDSIGSSFIKKNIDSIGSSFVKRPVDSLGSSFIKKNMDSIGS
SFIKRNMDSIGSSFIKKNIDSIGSSFVKRPVDSLGSAFIKKNMDSIGSSFIKKNMDSIGSSFIKKN
MDSIGSSFIKKNIDSIGSSFVKRPVDSLGSSFIKKNIDSIGSSFVKKGIDSIGSSFVKRPLDPIGSSF
VKKGIDSIGSSFVKKGIDSIGSSFVKRPLDPIGSSFVKKGIDSIGSSFVKRPLDRIGSSFVKRPLD
PIGSSFVKKGIDSIGSSFIKRQLDPIGSSFVKKGIDSIGSSFVKKGIDSIGSSFVKKGIDSIGSSFVK
RPLDPIGSSFVKKGIDSIGSSFVKKGIDSIGSSFVKRPLDPIGSSFVKRPLDPIGSSFVKKGIDSIG
SSFVKKGIDSIGSSFVKRPVDSLGSYFIKRGIDSIGSSFVKRPLDPIGSSFVKKGIDSIGSSFVKRP
LDRIGSSFVKKGIDSVGSSFVKRPLDPIGSSFVKKGIDSIGSSFVKRPVDSLGSSFIKKSIDSIGSS
FVKRSDDHHGRNNMKQRSFAAFKVFKSSRPKRLSQLRQRRSASGSVYTLGSKVHSLPELNR
SQRGRYLRLTIEDLESLLKYLEKQMYTRSEEEFGDFVDDPATGSDVIDN

>PEP3_M_leonina

MLSKSSLLSLAVCAILLVSQTLSQQSPSDNEVKQQLTSTATTDVHQKQRKTNKVLSMVTGGI
RGNAKGQQQQEPGEPVKNNEDKRTFGSSASKGFKKRSMDKIGHSSFAAFGKRPFDTIDGGS
AFSSFGKRPFDTISGNSAFARFGKRPFDAIDGGSAFSNFGKRPFDTISGNSAFSSFGKKRAFDSI
DGHSAFASFGKRPFDAIDGGSAFANFGKRPFDAIDGGSAFANFGKRPFDAIDGGSAFANFG

>PEP4_M_leonina

MHHVVEKPNLFPRVRSGNWSSSTKTTGQRDNMRPFGWPTVLKVLAVTQLVLMSSCIVQTA
SIEKVDGPTPESQKQPSAVGSLTDSKESPFVHAAHNEQADSLEETGSETSSNNDRSSASSNEP
LKEQVVNIQTLKSRLGSVRGSPSLSISSSYHKPSISDGRHELSKRSLDLLSSSRELSGMNQNFL
QPLQPRAENVGDKHVLYNRLVEVLRQLKVTLQNNRDKKSTGASLERPDLVDDDGAIDHSG
DFPKFVLFGSRNSGGDESPDGDGGSPAEGADFEEAEATDTPYSKRQDEISELKKRFDVLTNS
KMAGFNQNYLKKRDLAILAQSPMSGMDQNFMKKRFNYLSGGAISGFNQNHLKKKFDSLSS
GVMSFGQNHLKKRFDSLDSGLSSFNQNFIKKRLQPFMSSNLSAFNQNYLNKRFDSFSDSGLS
AFNQNYLEKKFDPFSSRGFTRFNQNNLKKKLNSLSNDGLSGFNQNHM

>Pleurin_M_leonina

MYQVLQLLTICIFAGSTCAVFYIPGSVVSYPKMGKRAFYTNATGNRYPLMGRRSSPSDREAQ
KEDGDAPEVVQESGEYNKRGVFTQGPHRSFPRVGRSGAQVSVNAKLRSDLLRSLAARLNDN
DLAEGPDSGPDSGRAVKAELAGQGERSGADNSERGIPLTQFLFLIMDEDGDGRLSKGEFSTG
MDKVMGQNLLC

>PRQFV_M_leonina

MNSSLLVSFLIAFLSPGLFLTHAVPSASLPLAQQKNSKDGIDPLPPVYRIEDRLTQHEPQGFYL
VDPLANEHEEDEDEDNNTSVDGSSENIPRGRLIGEPEEIPLKLLVSGLLGNDVHLQENLDFLK
SVGADKRAREFIGKRAREFIGKRAREFIGKRAREFIGKRAREFIGKRAREFIGKRNDDDVLLSI
GHELVRDRMYPIGAQERMDGKRAREFIGKRAREFIGKRAREFIGKRAREFIGKRAREFIGKRT
QEFIRKRNAGLEYEEPAKRAREFIGKRAREFIGKRAREFIGKRSDIEMDKRLREFIGKRSDIEM
E

>SCP_M_leonina

MEMTLPRATVSLTLLFVLICTVDAMNYLAFPRMGRSNYLAFPRMGRSGYAGFPRMSRSQVR
ADTDSQNSDCCGQGLKSEFSVSEDGKEELHSICTATIPVCCDGLKELNDEKPNGVVHTMCVP
YVSIFVNAEEKLKRLFSK

>Sensorin_M_leonina

MIPFYKGSQISLCYQTTRNQVEQGGRGQHGRVPFSVHVSHSVSGTGPLPGRSSLLIGQGQGTS
GDVIRQQQADDAHQNEACLNQGRPWSSGHDGLGCFVFLFLFFFPVFCYCCSCIFLEHVFLFP
SSLSGYVFLIYILIFFFYFSLLNKSLFCSVLISRL

>TemptinA_M_leonina

MRRILITPPVFPMSRSYSHFPIVYFVSVFAAVLLVPCGHAYPSFLEAIPNGRSVKHPCISFLKW
YGVGHTNPFGGGPKNPFGIDFAEAGYRWTAVLCAKDSDKDGDSNGQELGDPLCVWTPGKT
PSRTSDITHPGIKTGDLAGCFCPFAKLCPFPRS

>TemptinB_M_leonina

MKIVLAFTAMVAMATAFPGYQDAIPNGHSVPDPCNPGATWNGVGHVISRGTGDRNNFGNA
FAAEGHRWTVALCQADSDHDGKTNGMELGDPSCMWTPDNQSPLSPASSHPD

>Whitnin_M_leonina

MENHQSILLVILGSLLCASLVSCLPKRSAEDILQDTSGMVLDKRPKYMDTRRDLDVFKDLVL
MSLQELVDEGRLEPSTLPEEDRETSKPVEKRRYMGLCMHRQANQYIPFPCLRTGR

Table S3: Predicted prohormones from *P. californica.*

>Abdominal_ganglion_neuropeptides_L11_P_californica

MEPQAVLYRLLLVVLVVCVLLHPSSQGNGKGKRRRRLRINCAKYVFAPGCRGIAAKRGAPQ
TLSSLPSFSPMEEDSLLYKLLRDRQSESQENSQGSLPRLVMAAARLLKARPDSQNRFPRGLEY
QTRRRNREEPSAREEEEEEEENMEGEQFEEPSLENQLLQIWEQEPQGLQRP

>Abdominal ganglion neuropeptides L5-L67_P_californica

MASSNVIARTLALLFIVLSVCSAVPKWRPNGRMGKRTQPSLPFPLIEPDYRDSRLTEIPVEMF
ASPQELSQAGQKPRLCSVSGVSGYPQCEYSVLSQPAAGRP

>Abdominal ganglion neuropeptides R3-14_P_californica

MTPHLLLFCIRVPYVLLACTSCIFAGGGIRRPCGPFPGSCGIFHPPCNSLLYDQSPRKQNNLSH
ATFLFFFFPSSLS

>Achatin_P_californica

MAAIFILALVGFFFLLTLEFNTASQIQLSDPSALDLVDHFIETNQPMGKLGFVDKRGFVDKRG
FVDKREEAEKRGFVDKRDLAHKSGFVNRRGFVDKRGDLKFCRPREYRGSMLNMIIYSFGRI
GVKYTAVRWR

>AKH_P_californica

MNTNRLLLLSALVIVGLSTSSAQIHFTPGWGGKRSQLEEIDRTDRMSCFDQLDMEILMELSRL
IRKQAVKLGFCLKGCPTL

>Atrial_gland_and_califin_peptides_P_californica

FLVANQDRSIWAQHQRAEGGVREGSGEAVQASSDGLHPGELQHSDPDAPQPYAGQGERHP
TGVRMAGEEDGQEPLASLVLPVNQSDGQRVAQTLQHCGAGAHFPERLQPEIRGPIKILEQSV
NRPVKVFGQVGLLLSLWQGRACQTRQLPRQK

>Buccalin_P_californica

VGKRGVDPFSFASGVGKRGVDPFSFASGVGKRGVDPFSFASGVGKRGVDPFSFASGVGKRG
VDPFAFASGVGKRGVDPYAFISGVGKRGVDPFAFASGLGKRRVDPFAFASGVGKRGVDPFA
FASGVGKRGVDPYSFVSGVGKRGVDPFSFASGVGKRGVDPFAFASGVGKRGVDPYSFVSGV
GKRSAEEFASDEDKTDIEDSSSEEKDSEDSSLDAADSEKRDVDTYSFAPGIGKRHIDHFGFSS
GIGKRKMDHFGFNAGIGKRLDQFGFNAGIGKRRIDNFGFNAGIGKRRMDNFGFNAGIGKRRI
DNFGFNAGIGKRRMDHLGFASGIGKRSARDSQSLEDSDDSHSIDHAVTKKRSASQVSEST

>Cerebral peptide_P_californica

MKAINAINASQIPTLLLQLALVAAILLIDLSHSDDSTEAFAASKRATSSDLSSSRHKRAPGWG
KRDSVYRNDPVGDFSRWSNKIAEVSDAQGRSKALGRLNSKMLSSLADVPLKRAPGWGKRT
VESGEYSCQIMQRAERAGK

>Cerebrin_P_californica

MCSCKIVMLFVLVVALSLVVSEISAAPYFAARSPMEMIRDNRGPFGSVSNVGSRALSNFLKR
NGGTSDAMYNLPQLMEIGRR

>Enterin_P_californica

LVGQSSSLAQDDADDRRNYDELDDLPKAEEGETLDDKRGFEHAFVGKRPFEHAFVGKRPFE
HAFVGKRAFEHAFVGKRDTGFDHAFVGKRPFEHAFVGKRPFEHAFVGKRGFEHAFVGKRPF
EHAFVGKRDTGFDHAFVGKRPFEHAFVGKRPFEHAFVGKRPFEHAFVGKRQFEHAFVGKRP
FEHAFVGKRGFEHAFVGKRPFEHAFVGKRGDFEHAFVGRSSDDQLEEGSSESPDEVLAKYL
DDAVGNKLDEKRAFEHAFVGKRAFEHAFVGKRGFEHAFVGKRPFEHAFVGKRPFEHAFVG
KRPFEHAFVGKRPFEHAFV

>Enticin_P_californica

TAKGRVFSTPGSQSPSLLRATLTLEARQTLQGTRDCATINWGLRRARMSSFTTTQQP

>FMRFa_P_californica

MTSLCCLALTALLALQVSSHNARAQSVICDDPELCLPKRFLRFGRSGSESSDGYYLTPHLAFS
SGRRFVRLGRAYEPYQDKRFLRFGRASAGGQRSEESLLREALMQAEEPLYRKRRSAQQDEA
DEAEEGAAEGAQQRMKRSPAEEEEKSEGAIKALQKRDVGGGSAAGDAEEEDIISRQILGLGG
GQVGESGDVIDGFRKRFMRFGRVPGDEHEEAEKRFMRFGKRFMRFGRQAGKRFMRFGRGL
ESAKRFMRFGRPSNAALEGLEGEKRFMRFGKRDTKAVESRTGADSQ

>FCAP_P_californica

MATRLSLKLVALLLVCMAGPWSEVNASRWPLDKIGAGELGIFSQMVADRLRDGDDTAGLL
YLPAKRHASKVKMYSIPDDLSEVRYVLQGASALASPNKRVFDSLGGYEVHDGKRSYETQR
WKRSVDTTEEHAEDNESEQRTKRDDNVQKRGLDSLGGFNVHGFKRGLDSLGGFNVHGWK
RDDADKRGLDSLGGFNVHGWKRGLDSLGGFNVHGWKREDGEKRGLDSLGGFNVHGWKR
GLDSLGGFNVHGFKRDGEKRGLDSLGGFNVHGFKRDGEKRGLDSLGGFNVHGFKRDGEKR
GLDSLGGFNVHGWKRGLDSLGGFNVHGWKRGDDEKRGLDSLGGFNVHGWKRNGEVEDA
LLDLSSLSENGEKAVDGLKRGLDSLGGFNAHGWKRSVEQAVPTTDDMSN

>FMRF-related_neuropeptides_P_californica

MTSLCCLALTALLALQVSSHNARAQSVICDDPELCQLTQAAQPPKSGQSQQAWTLPSRFHRP
NRDAEEDDGVEEEDPYLRFGREADPLAQARYARSGDIYPRYGRSSDQKTIRVRRGDPFLRFG
KGDPFLRFGKGDPFLRFGKGDPFLRFGRSDPLMRFGRGDPFLRFGKGDPFLRFGRGDPFLRF
GRGDPFLRFGKGDPFLRFGRGDPFLRFGKGDPFLRFGRGDPFLRFGKGDPFLRFGRSDISMDS
KKRSEMDEDTFVDRSNPMVGGQRKKRSLPDQQATSSGLGNQASDSMSPFAGMSVSRRADV
YPVFGKRLAHVLGLQQESTARVGGYGTPG

>GNRH_P_californica

MLTYAKTTGSPSPTKAATLALLVVLSLLLASSNAQSYHFSNGWFPSGKRASGAGTGAGQGD
VVRRLIKATTVGDDACSFRPDLMEIVNKLVQDEIARIQASCLF

>Insulin_P_californica

MSQNWLTQHAIQLCVVIVLLTLASHTRYSQASFEHTCTSIDAPRRGVCGPDLVELISTLCRNY
AIFSGGRQRRSADKLSLDSRLKRVLLNKKDAFSYLDKRDYGSSGITCECCYNQCSIREMISYC
LLQAVDDSGRSNATY

>LFRFa_P_californica

MENSLNTFGAILLLALFASVCCKETAQMSDASQESNHEKLHQAQKRSAILEPTETESGLSSD
DEANLLSYLGAPYLHPVDSDKRSSLFRYGKRGGSLFRFGKRGSLLRYGKRGSFSQEGKRGG

TLFRFGRSGVSQEDAMPRIQRTLFRFGKRSDLDSDAREFDGYMRVEPSKRGDSFHWGLDSD
E-RIEEENFFLKQELRSIRILRALLVLVCPI

>MIPR_P_californica

LWNRDGWTHSTQSGRQTKPTETGRRRETNQASCRSLSLSLSAGKKAPSISIPQANKRFCGNR
RGIGRLQQKTSIRVKQFWSYHRRSNFESVQSSKCNRRLDLTRATSSSSITMATQHVQITFLVL
VIAFVAGTLTTDDLSEDTAAHQLQRRDTLNSDSDSSAHARHESERWKRGTSSESEEADIHSV
AKRATEFLSKTRHRRTPLIARSQYLDLLDRDADEDESMLSDEEDDGYFNSDSLVDDAETRD
MQKRRSPWFVGRRGAPLFVGRRGAPLFVGKRDAPPFVGRRGAPLFVGKRHGPAIVSRRDAP
PFVGKRGAPLFVGRRGAPLFVGRRGAPPFLGRRSIVDLSGLPEEEANEQIERRRKQRGRWLC
KTACFLFTKSPLVFHTFKIWLLYGYLVMGLHFSPHFSKPFCDQLFFLCIRKRFLQSGVGEFGF
SIFCDRKLES

>Myomodulin_P_californica

MDPTRHCLLLLLVLLCHSGWAKESASSSDSSQQELSSQSGASSRTKRQSYSMLRLGRGLQ
MLRLGKRGRSDLYSPVDADSSPLLDNAYYSSFPDRLEDFPSSRLRRSTMNPEVDAEPAVEPG
VVDGETGSEVDGYYVLPDDFLEELLEEMDTEGLTEEQKRSLSMLRLGKRADFIPVDEVDGP
EAEKRNLQMLRLGKRNLNMLRLGKRNLNMLRLGKRNLAMLRLGKRNLNMLRLGKRNLN
MLRLGKRNLAMLRLGKRNLQMLRLGKRPYDADTPEKRTLHMIRLGKRPEDEKRNLNMLRL
GKRQADKKNFQMLRLGKRDGELEAPREAKSLDRLRLGKRSAQ

>Myomodulin2_P_californica

MTTLFVSSLCYAVLVFCFYVCTARAKTTSSAEASGDSSAEFNKRAWNMLRLGRGLQMLRL
GKRSIDSSSENTAAPSRSARSVDSSEKSRHSLWTDAEFRDMLAALMGEARDETRRQPPLPRY
GRDSDRGLMELLADADGGFITFRPAPRGGRYRRSVSGASSGIPVFVGSYFPEEFKEQMTRAIP
RPRIGRLVENDEQEFDDKLYSRSIPRPRIGRFLQNVAHVETKAIPRPRLGRGKDSESSQ

>NdWF_P_californica

MARLVAATCLLLVLLGCLSTSTVHANWFGKRGDQDDLFHMLLQQPDSMSTRDSLDAEAAI
MAVERIVSQWRQHRRAKMASTVQE

>Neuroactive_polyprotein_R15_P_californica

MARLVAATCLLLVLLGCLSTSTVHANWFGKRGDQDDLFHMLLQQPDSMSTRDSLDAEAAI
MAVERIVSQWRQHRRAKMASTVQE

>NPY_P_californica

MHKFLLLALLLASLATLQISAMEAMLSPPVRPKEFKSPAELRRYLKALNEYYAIVGRPRFGK
RNNVENRMDEWYNTNRDVDRLSSWDTLE

>Neuropeptides_CP2_P_californica

MDLSQMMCLILSTLLMASITLSSAYPYGSQGSYLEDEDFPQFLFDVDSRQHVPSPSEKFFFKD
SKDAASPRSKKSMNFGFAGIDNLDAITSVLNRSKAKPRSGPVGRSNMKYLLSDNLRERMPSI
GWI

>PEP1_P_californica

MTAMKIAVLAFLLLCVFAQTKEAKEIVENDQGSASTSLEKRAKRPHDSISGHDGLMGFAKR
PHDSISGHDGLMGFAKRPHDSISGHDGLMGFAKRPHDSISGLDGLMGFAKRPHDSISGHDGL
MGFAKRPHDSISGHDGLMGFAKRPHDSISGHDGLMGFAKRPHDSISGHDGL

>PEP2_P_californica

MVTKVTCLPWKMTCLLVLSALANAGFSKANEIKDSNRRPSSLHAQHTHFSHSRDAFNRPGS
WKHRREFYQELNQHPSAYTGSHSQVAGQPERPFPADQRPGQAGATGHRVMTHFASLADQR
PSKRYLDNLANSLIRRQGPGSASAANRNQGVDKRYLDELASSLIKRKSDGTDKRYLDSLANS
LVKKDSTDKRYLDSLANSLVKKESHDKRYLDSLANSLVKRESHEKRYLDSLANSLVKRDSK
DKRYLDSLANSLVKKDSHNKRHLDSLANSLVKREPKDKRYLESLANSLVKKESKSKRHLDS
LANSLVKKESGNKRYLDSLANSLVKRDDKDKRHLDSLANSLVKKESDKRYLDSLASSLVKK
ESDKRHLDSLANSLVKKESDKRYLDSLASSLVKKESDKRYLDSLANSLVKKVSDKRHLDSL
ANSLVKKESDKRHLDSLANSLVKKESDKRYLDSLASSLVKKESDKRHLD

>PEP3_P_californica

MGHPLQLLIFLAMCSYAVGAEEQNNKDTPHSTSSQVEKRSYEADSSGQSEKRRLDSIGNSAF
ASFGKRENDPSSPSTDKRRLDSISGSAFGSFGKREQDTSSPDLDKRRLDSISGSAFGSFGKREQ
DASSPDLDKRRLDSISGSGFGSFGKRKFDSITGSAFNTLGKRRLDSITGSGFNGFGKRRLDSIT
GSGFNGFGKRRLDSITGSDFNGFGKRRLDSITGSDFNGFGKRRLDSITGSDFNGFGKRRLDSIT
GS

>PEP4_P_californica

MSPFRQAKRHLISRYKLVLLLLVTFSLLNSKCLADGNDQVKLKQQDSSTSPSSPEENKQALQ
SLDHSHSADDASAINTDTSLPKTASSGEGKVQESINEINSLSSSETTVSSLLSVNEKQKSSPVS
GSEVGTSGSSEEESGSHHLSRRSFDIVGEAGDGDGNDGGVSQEEMKALYNRLGRIAERMKD
SSESDEKEEDMEFSDEFEGDGSQEQDDNERDDSGSVGSDLDSMPSDDNVDEGDYVDKRQFD
SINDGRLNGMSQNFLAKRNFDSISRGLVSGLNQNFLGGKRQTFPNMGYENYSPYYLRKRQF
DSIGAGPMSGMHQNYLGKRYFDSISHGKMNGLRQNFLHKRQLDSIGAGMVSGLHQNFLGR
RNYITPRMSNGFLSNGFDKRFDSISSGRLNGFNANFLGKRDYDDFFGAYDRLNKRQFDSIAT
GRLSGLGQNFMHKRQFDSIGTGTFGGLQQNFLDDKRRFDSISSGHMGGLNQHFLGKRDGGD
QGNMETKRQFDSIGTGKTSGMGQVFIGKRQFDSIGSGKVDGLHQNFLGKRLDSTSAGSISGT
HRNFLGKRSFDSIHSGKIDGLNQHFLGKRQMDSIGSGRLDGLNQNFLGKRQFDSISSGRMDG
LHQNFLNKRFDSIDAGKLDGFHQNFLGKRQFDSIGAGHLDGLHQNFLGKRPFDSISSGRTDG
LHQNFLGKRFDSISNGKLDGLHQVYLGKRFDSISSGKLEGLHQNFLGKRFDSIGNGHLDGLH
QNFLGKRFDSIGTGKLAGLHQNFLGKRFDSIGTGKLAGLHQN

>Pleurin_P_californica

MFMVVLTGSSCAVFYTKDPEVDYPRIGRRSFYTTSEGNHYPRIGRRDVGAMSSLLPGSEFMD
YSDLSKGNHGTMNKRGFLPNIGRRASLSNQNDLHYVLGGSKKKEFLSNLAANSKSDNLRGE
SEEKSTADDFELERLNVPLDFLFIAFDLNGDGKLSKSEFVTGMQVYKQQPRQS

>PRQFV_P_californica

KRPRQFVGKRDDDLEQFLAEKRPRQFVGKRDDEEDIFAEKRPRQFVGKRDDGEGDILAEKR
PRQFVGKRPRQFVGKRGLDLLAAEKRPRQFVGKRDPADLFLENKRPRQFVGKRPRQFVGKR
FDDFFSQEKRPRQFVGKRFFDDDMLSADKRPRQFVGKREFDDIFDAEKRPRQFVGKRQFTFD
DDFFRQDKRPRQFVGKRFSDGDLFSQDKRPRQFVGKRPRQFVGKRPRQFVGKREFEDGLFL
ESKRPRQFVGKRDFGDDLFLEGKRPRQFVGKRDLDGGLFAADKRPRQFVGKRDFGGDFML
EEKRPRQFVGKREFDDGFLLADKRPRQFVGKREFDVDFLEADKRPRQFVGKREFDDDFLEA
DKRPRQFVGKRDSNDLLSALEKRPRQFVGKRDFSEEGIFLEDKRPRQFVGKRSVDATSPQSA
ESVSKRSAESSDELSSSSSSAAEIANSHVSSLVKRDDHHVSDNQEQAVEKRNVRSASETDQT
GQSLR

>Sensorin_A

MSSPSLPSKTASLLLLLLALCLLLNDVTCAPKLRPMRRGQRKSVRTRFRMGYMFGKRSMDV
SASQTTPLFDTITKQLMTKQGLAAILRQEPSKIDEAVNFLDRDNNGFITVSDLL

>Temptin_P_californica

MDKTALGFVSLLLLAIISPALGYYAFQVEIPNGNNVEHPCKVNYQWRGVGHKNPLGGGTRN
VFGRDFKNAGSKWTKELCNKDSDGDGMTNGEELGDPECIWVKGQKPQRITEITHPGICNPF
GVGKCEGLDTWVDCKVETMTDCDKIKEPDVKALELRFPNTTIPDKETNYFCMTFDLPHLED
YHIVAYE

>Whitnin_P_californica

MEYRVAHLFTFFMLLIAVSMVSCLPTRSAADEALQDVSGMAVGKRPKYMDTRRDLDVFKD
LVLASLQELVDENKVNPSILVQDDEEQTVEKRRYMGLCYHQKANTFVPFPCLRSGR
